# Awareness, knowledge and attitudes of human papillomavirus infection, screening and vaccination: a survey study in Greece

**DOI:** 10.1007/s00404-024-07398-1

**Published:** 2024-03-08

**Authors:** Vasilios Pergialiotis, Dimitrios Papageorgiou, Athanasios Douligeris, Anastasia Mortaki, Dimitrios Efthymios Vlachos, Nikolaos Thomakos, Alexandros Rodolakis, Dimitrios Haidopoulos

**Affiliations:** 1st Department of Obstetrics and Gynecology, Division of Gynecologic Oncology, Alexandra Hospital, National and Kapodistrian University of Athens, Vasilissis Sofias Avenue 80, Athens, Greece

**Keywords:** Human papillomavirus Infection, HPV awareness, HPV vaccination, HPV screening

## Abstract

**Purpose:**

To evaluate the awareness and existing knowledge of a portion of the Greek population about prevention, screening, and HPV vaccination.

**Methods:**

A questionnaire designed in Google forms has been distributed through social media between June 2021 and December 2021 in men and women aged > 16 years old. Statistical analysis was performed using the SPSS 20.0 program. Inferential analysis was performed to evaluate differences in responses among men and women.

**Results:**

We enrolled 2685 participants. Of those, 2285 were women, 386 were men, while 14 respondents chose not to respond to this question. Various age groups were detected with those aged between 26 and 30 years old being the predominant one. Participants with a higher education constituted 36.5% of the population. Most respondents were married (59.8%). In socioeconomic terms 75.5% of participants were employed whereas, monthly income ranged between 1000 and 1500 euros in the predominant group (36.8%). Only 40% of females and 3.9% of males were vaccinated against HPV. Adolescent immunization, acceptability rates reached 92.7% among female and 82.1% among male responders. Although, only a small proportion of the participants were not aware of the existence of HPV, 24.1% of males and 23.4% of females had the impression that condom use may provide absolute immunity to HPV and only 51.6% of males and 60.4% of females were aware about the high prevalence of HPV in the general population. Logistic regression analysis indicated that male participants as well as those aged > 50 years and those choosing to reject vaccination had decreased knowledge of the basic pathophysiology of HPV infection, as well as knowledge related to the existence and use of HPV DNA as a screening tool and the existence and efficacy of HPV vaccination.

**Conclusion:**

Our results indicate that although awareness of the existence of HPV infection is high in Greek general population, the actual perception of the pathophysiology of transmission and importance of HPV testing and vaccination is low. Targeting specific population groups is essential to help increase HPV coverage and screening.

**Supplementary Information:**

The online version contains supplementary material available at 10.1007/s00404-024-07398-1.

## What does this study add to the clinical work

**Table:** 

The present study is the first to highlight the beliefs and knowledge of the general Greek population regarding HPV infection and vaccination. In fact, this study coincides with the inclusion of HPV vaccination in the national vaccination program for males as well and therefore can be a tool for better information and prevention.	

## Introduction

Human papillomavirus (HPV) is the most common sexually transmitted infection (STI) leading to a significant number of benign, premalignant, and malignant lesions. In particular, persistent infection has been implicated as a causative agent of multiple malignancies such as cervical, vaginal, and vulvar cancer in women, penile cancer in men as well as anal and oropharyngeal cancer in both sexes [1, 2].

The scientific advances that have been made in recent decades in the understanding of HPV infection are unfortunately associated with a corresponding lack of patient information on the prevention and screening practices. Although, in Greece, HPV vaccination program has been instituted in 2007, vaccination against HPV of young adolescents is not adequately recorded while it is generally estimated that vaccination coverage rates remain very low [3, 4]. Specifically, between 2008 and 2014, the average vaccination coverage of Greek teenagers (aged 11–16 years) was just 8.9%, rising from 3.2% in 2008 to 17.1% in 2011. However, it decreased in 2012 to 2.1% and reached 9.2% and 11.5% in 2013 and 2014, respectively [5]. Furthermore, despite various endeavors, there is presently no national cervical screening program in Greece, and most women are examined on an opportunistic basis [6].

The aim of the present study is to evaluate the existing knowledge of a portion of the Greek population about prevention, screening, and HPV vaccination. Evaluation of the perception of the utility of cervical smear and HPV DNA will be performed as well. The findings of this study aim to serve as a pilot for the construct of further actions that will help promote HPV awareness on the basis of a constructed public health strategy.

## Materials and methods

### Ethics information and survey distribution

The survey we conducted was an open survey, designed in Google forms and distributed through social media (Twitter, LinkedIn, Facebook) between June 2021 and December 2021 in men and women aged > 16 years old that were able to read and comprehend read language. A campaign took place in three major hospitals in the region of Attica (including Alexandra University Hospital, Athens Naval and Veterans Hospital, Iaso Hospital) and the survey was communicated using the above mentioned social media through the personal profile of obstetricians-gynecologists and midwifes. The study was approved by the Institutional Review Board and Ethics Committee of our hospital and participants were informed that all information were anonymous and provided their consent during the first step of the survey.

### Survey description

The survey was designed taking into consideration previous studies that performed surveys related to HPV-related awareness [3, 7, 8]. The questionnaire was pre-tested in a group of 50 obstetrics and gynecology residents that confirmed the ease to use of the electronic platform as well as the ease of understanding the questions included. Overall, the survey included 24 questions relevant to the prevalence and pathophysiology of HPV infection, 5 questions that aimed to evaluate knowledge related to the existence of HPV testing and 9 questions that evaluated knowledge related to the existence and efficacy of HPV vaccination.

Multiple choice questions were avoided to help minimize the possibility of participant misleading. The ability to refuse to respond was provided in each question to avoid forced responses such as “I do not know” [9, 10]. Overall, the potential responses to the distributed questions were TRUE/FALSE, YES/NO, Do not know, Do not wish to answer this question. Our questionnaire was in total five pages long and each page consisted of ten items. The questionnaire used in the framework of the present study is included in Appendix.

Every individual, who accepted to participate in our study, completed the survey by answering the last item of the questionnaire. Hence, the completeness rate is calculated to be 100%. The response rate in our case could not be calculated, since our study was an open survey, accessible for each visitor of the sites that it has been uploaded.

### Statistical analysis

Statistical analysis was performed using the SPSS 20.0 program (IBM Corp. Released 2011. IBM SPSS Statistics for Windows, Version 20.0. Armonk, NY: IBM Corp.). Inferential analysis was performed to evaluate differences in responses among men and women using the Chi-square test and Fisher’s exact test. The number of false responses was documented per individual that participated in the study and the cumulative median value as well as the interquartile range was documented separately for men and women. Logistic regression analysis was performed to evaluate individual characteristics that predisposed participants to respond in the lowest quartile of correct answers following exclusion of questions that were considered redundant in the results of the Rasch analysis as explained later. The level of significance for all analyses was set to *p < 0.05.*

Rasch analysis was performed using the Jamovi software to evaluate the reliability and validity of the questionnaire using the methodology that has been previously described by Anagnostou et al. [3]. The analysis was performed separately for males and females and person reliability index was calculated in both cases as significant differences were observed in the rates of answers among men and women in the univariate analysis (as explained in the results section). A cumulative (per subgroup) person reliability index above 0.75 was considered as adequate to denote a reliable evaluation of individual reliability indices (meaning that persons with high ability in detecting correct answers are correctly identified). Local independence of included questions was evaluated with correlation coefficient analysis of residuals and values that ranged between − 0.300 and 0.300 were considered as indicative of independence among questions included.

Calibration of the difficulty of each question was performed and infit and outfit mean square standardized residual (MNSQ) values were evaluated. Values that exceeded 0.7 and 1.3 were considered redundant and the results of relevant questions were considered as potential noise. Question difficulty was evaluated using calibration of item difficulty per subgroup of participants (men/women) and a summary plot participants’ ability to identify correct answers was also constructed.

## Results

### Baseline characteristics of participants

Overall, 2685 answers were received within a period of 6 months. Of those, 2285 were women, 386 were men, while 14 respondents chose not to respond to this question. Various age groups were detected with those aged between 26 and 30 years old being the predominant one, followed by those aged 31–35 years. Participants with a higher education (university) constituted 36.5% of the population, whereas participants with a Master of Science (following university or college) constituted the second more common group (25.5%). Most respondents were married (59.8%) as opposed to unmarried (34.5%) and divorced (4.4%). More than 2,500 participants had completed sexual intercourse (95.9%). In socioeconomic terms 75.5% of participants were employed whereas 9.5% of them were unemployed and 12.8% were university students. Monthly income ranged between 1000 and 1500 euros in the predominant group (36.8%) which was followed by the group with a monthly income between 500 and 1000 euros (19.3%).

Only 40% of females were vaccinated against HPV, a percentage that is significantly smaller to that observed in other high-income countries. The percentage of vaccinated males was extremely low as only 15 individuals reported that they were vaccinated (3.9%). Analyzing the perception of participants concerning adolescent immunization, acceptability rates reached 92.7% among females and 82.1% among males that responded. All the baseline characteristics of the participants are briefly presented in a Table in Appendix.

### Analysis of knowledge items related to the pathophysiology of HPV

Table 1 summarizes the correct answers per individual knowledge item among males and females that participated in the survey. Significant differences were noted in the majority of questions with females responding better than males. Of note, 9.1% of males were not aware of the existence of HPV. A large proportion of participants (24.1% for males and 23.4% for females) had the impression that condom use may provide absolute immunity to HPV. Only 51.6% of males and 60.4% of females were aware that the prevalence of HPV in the general population (on a lifetime basis) is high. Interestingly, we observed that approximately 10–13% of participants believed that the infection may predispose to HIV infection. Whereas a large proportion was aware that HPV predisposes to cervical cancer only half of participants knew that the infection is also related, although less commonly, with head and neck and penile cancer.

**Table 1:** Univariate analysis of differences in responses among men and women

	Men			Women			*p* value
	Yes (True)	No (False)	Do not know	Yes (True)	No (False)	Do not know	
*Awareness of HPV infection and its pathophysiology*							
(1) Awareness of HPV infection	351 (90.9%)	35 (9.1%)	–	2230 (97.6%)	55 (2.4%)	–	< .001
(2) Awareness of multiple subtypes of HPV virus	288 (74.6%)	94 (24.4%)	4 (1%)	1989 (87%)	284 (12.4%)	12 (0.5%)	< .001
(3) Awareness of HPV transmission with sexual contact	362 (93.8%)	3 (0.8%)	21 (5.4%)	2215 (96.9%)	10 (0.4%)	60 (2.6%)	.037
(4) Perception of HPV transmission exclusively with sexual contact	70 (18.1%)	213 (55.2%)	103 (26.7%)	585 (25.6%)	1367 (59.8%)	333 (14.6%)	< .001
(5) Perception of infection of women exclusively	16 (4.1%)	334 (86.5%)	36 (9.3%)	47 (2.1%)	2129 (93.2%)	109 (4.8%)	.005
(6) Perception of immunity of men in against HPV	20 (5.2%)	332 (86%)	34 (8.8%)	46 (2.0%)	2117 (92.6%)	122 (5.3%)	< .001
(7) Perception of complete immunity when condom is used	93 (24.1%)	239 (61.9%)	54 (14.0%)	535 (23.4%)	1520 (66.5%)	230 (10.1%)	.023
(8) Perception of HPV infection as a rare incident	14 (3.6%)	310 (80.3%)	62 (16.1%)	10 (0.4%)	2155 (94.3%)	120 (5.3%)	< .001
(9) Perception of visible lesions in all cases infected by HPV	34 (8.8%)	274 (71%)	78 (20.2%)	65 (2.8%)	1999 (8.5%)	221 (9.7%)	< .001
(10) Perception of a correlation of the incidence of HPV with the lifetime number of partners	339 (88%)	10 (2.6%)	37 (9.6%)	1919 (85%)	174 (7.6%)	192 (8.4%)	.002
(11) Perception that asymptomatic carriers do not transmit	25 (6.5%)	295 (76.4%)	66 (17.1%)	61 (2.7%)	2008 (87.9%)	216 (9.5%)	< .001
(12) Awareness that most men and women will become infected in a lifetime basis	199 (51.6%)	70 (18%)	117 (30.3%)	1380 (60.4%)	317 (13.9%)	588 (25.7%)	.024
(13) Perception that HPV may predispose to HIV	50 (13%)	250 (64.8%)	84 (21.8%)	248 (10.9%)	1614 (70.6%)	403 (17.6%)	.001
(14) Awareness that HPV infection may cause cervical cancer	320 (82.9%)	6 (1.5%)	59 (15%)	2124 ((93%)	20 (0.9%)	134 (5.9%)	< .001
(15) Perception that HPV may be treated with antibiotics	66 (17.1%)	184 (47.7%)	135 (35%)	351 (15.4%)	1248 (54.6%)	668 (29.2%)	.003
(16) Awareness that HPV infection may be transmitted orally	269 (69.7%)	33 (8.5%)	82 (21.2%)	1750 (76.6%)	136 (6.0%)	385 (16.8%)	.001
(17) Awareness that HPV infection may be transmitted rectally	284 (73.6%)	24 (6.2%)	76 (19.7%)	1809 (79.2%)	68 (3.0%)	392 (17.2%)	< .001
(18) Awareness that HPV infection may cause head and neck cancer	201 (52.1%)	29 (7.5%)	154 (39.9%)	1286 (56.3%)	149 (6.5%)	829 (36.3%)	.031
(19) Awareness that HPV infection may cause rectal cancer	198 (51.3%)	21 (5.4%)	167 (43.3%)	1259 (54.7%)	125 (5.5%)	895 (39.2%)	.014
(20) Awareness that HPV infection may cause penile cancer	180 (46.6%)	30 (7.8%)	176 (45.6%)	1095 (47.9%)	172 (7.5%)	1005 (44%)	.063
(21) Perception that HPV may predispose to HSV	116 (30.1%)	123 (31.9%)	144 (37.3%)	732 (32%)	609 (26.7%)	924 (40%)	.025
(22) Awareness that HPV infection may cause papilloma	298 (77.2%)	12 (3.1%)	73 (18.9%)	2039 (89.2%)	37 (1.6%)	203 (8.9%)	< .001
(23) Awareness that HPV infection usually goes away on its own	40 (13.2%)	265 (68.7%)	74 (19.2%)	263 (11.5%)	1699 (74.4%)	293 (12.8)	< .001
(24) Awareness of what cervical cancer is	339 (87.8%)	47 (12.2%)	–	2230 (97.6%)	55 (2.4%)	–	< .001
*Awareness of HPV DNA testing and its utility*							
(25) HPV DNA test may be performed to detect oncogenic subtypes	166 (43%)	21 (5.4%)	199 (51.6%)	1359 (59.5%)	98 (4.3%)	828 (36.2%)	< .001
(26) Women with a positive HPV DNA test will definitely develop cervical cancer	16 (4.1%)	225 (58.3%)	145 (37.6%)	62 (2.7%)	1711 (74.9%)	512 (22.4%)	< .001
(27) HPV DNA test is performed to evaluate if vaccination is needed	56 (14.5%)	145 (37.6%)	185 (47.9%)	164 (7.2%)	1380 (60.4%)	741 (32.4)	< .001
(28) Papanicolaou smear I used to detect the presence of HPV virus	149 (9.1%)	87 (22.5%)	150 (38.9%)	1477 (63.6%)	543 (23.8%)	265 (11.6%)	< .001
(29) HPV DNA test can be performed together with Papanicolaou smear	124 (32.1%)	17 (4.4%)	245 (63.5%)	1093 (47.8%)	146 (6.4%)	1046 (45.8%)	< .001
*Awareness of HPV vaccination and its benefits*							
(30) A vaccine exists that protects against HPV infection	15 (3.9%)	300 (77.7%)	71 (18.4%)	78 (3.4%)	2052 (89.8%)	155 (6.8%)	< .001
(31) HPV vaccination is indicated only after the onset of sexual activity	69 (17.9%)	226 (58.5%)	91 (23.6%)	163 (7.1%)	1895 (82.9%)	227 (9.9%)	< .001
(32) Vaccinated women are 100% protected against cervical cancer	31 (8%)	209 (54.1%)	146 (37.8%)	218 (9.5%)	1615 (70.7%)	452 (19.8%)	< .001
(33) HPV vaccinated women may discontinue routine cervical testing	13 (3.4%)	285 (73.6%)	88 (22.8%)	29 (1.3%)	2190 (95.8%)	66 (2.9%)	< .001
(34) Vaccination prevents infection from all HPV subtypes	37 (9.6%)	175 (45.3%)	174 (45.1%)	149 (6.5%)	1469 (64.3%)	667 (29.2%)	< .001
(35) Vaccination against HPV may prevent several other STDs	14 (3.6%)	286 (12.3%)	86 (22.3%)	51 (2.2%)	2038 (89.2%)	196 (8.6%)	< .001
(36) Only one dose of vaccine is needed	34 (8.8%)	103 (26.7%)	249 (64.5%)	167 (7.3%)	1355 (59.3%)	763 (33.4%)	< .001
(37) HPV vaccination is more efficacious if performed prior to the onset of sexual activity	138 (35.8%)	60 (15%)	188 (48.7%)	1384 (60.6%)	217 (9.5%)	684 (29.9%)	< .001
(38) A vaccine exists that protects against genital warts	122 (31.6%)	41 (10.6%)	223 (57.8%)	878 (38.4%)	252 (11.0%)	1155 (50.5%)	.028
Have you received an HPV vaccination?	15 (3.9%)	371 (96.1%)	–	914 (40%)	1371 (60%)	–	< .001
Do you believe that adolescents should receive HPV vaccination?	317 (82.1%)	69 (17.9%)	–	2118 (92.7%)	167 (7.3%)	–	< .001

Response was not applicable as the questions provided binary responses (Yes / No, true / false)

In terms of participant baseline characteristics male sex, age groups of > 50 and 21–25 years old, serving as a private employee or self-employed and non-vaccinated status as well as not favoring adolescent vaccination were identified as statistically significant factors that were associated with an increased rate of incorrect responses (Table 2).

**Table 2:** Binomial logistic regression analysis for the estimation of the odds of incorrectly responding the questionnaire

Variable	HPV awareness		HPV DNA testing awareness			Vaccination awareness	
	OR (95% CI)	*p* value	OR (95% CI)	*p* value	OR (95% CI)		*p* value
Sex (male ref)	–	–	–	–	–		–
Female	1.76 (1.30, 2.37)		2.79 (2.07, 3.77)	< .001	2.65 (1.97, 3.57)		< .001
Age group (31–40 years ref)	–	–	–	–	–		–
41–50 years	1.38 (1.01, 1.90)	.047	1.22 (0.88, 1.71)	.239	1.13 (0.82, 1.55)		.448
> 50 years	3.30 (2.09, 5.19)	< .001	1.16 (0.71, 1.88)	.551	2.16 (1.36, 3.42)		.001
26–30 years	1.25 (0.92, 1.68)	.150	1.33 (0.97, 1.81)	.073	0.67 (0.49, 0.91)		.010
21–25 years	2.33 (1.31, 3.37)	< .001	2.34 (1.58, 3.45)	< .001	1.00 (0.67, 1.49)		.995
16–20 years	2.93 (0.76, 11.35)	.119	2.99 (0.72, 12.41)	.130	0.98 (0.21, 4.59)		.975
Education level (University, PhD ref)	–	–	–	–	–		–
College	1.20 (0.89, 1.62)	.243	1.20 (0.87, 1.65)	.246	1.10 (0.80, 1.52)		.543
Basic	0.93 (0.66, 1.31)	.666	1.88 (1.28, 2.76)	.001	1.88 (1.28, 2.77)		.001
Family status (Married ref)	–	–	–	–	–		–
Unmarried	1.14 (0.37, 3.54)	.821	0.33 (0.12, 0.95)	.040	0.72 (0.24, 2.20)		.563
Sexual activity (started ref)	–	–	–	–	–		–
Not started	2.73 (1.32, 5.61)	.006	1.64 (0.77, 3.45)	.201	1.66 (0.76, 3.61)		.200
Parental status (parents ref)	–	–	–	–	–		–
Not parents	1.00 (0.69, 1.45)	.983	1.23 (0.84, 1.81)	.289	1.28 (0.88, 1.87)		.192
Employment (employed ref)	–	–	–	–	–		–
Unemployed	0.93 (0.45, 1.72)	.808	0.51 (0.26, 1.00)	.051	0.68 (0.34, 1.36)		.278
University student	0.85 (0.47, 1.55)	.597	0.51 (0.25, 1.04)	.065	2.09 (1.12, 3.89)		.020
Working position (Public employee ref)	–	–	–	–	–		–
Private employee	1.62 (1.23, 2.13)	.001	1.48 (1.12, 1.97)	.007	1.96 (1.48, 2.60)		< .001
Independent employee	1.59 (1.11, 2.26)	.011	1.44 (0.99, 2.08)	.053	1.81 (1.26, 2.61)		.001
Income (1000–1500 euro ref)	–	–	–	–	–		–
1500–2000 euro	0.90 (0.59, 1.37)	.616	1.03 (0.68, 1.57)	.885	0.89 (0.59, 1.35)		.585
> 2000 euro	0.81 (0.49, 1.35)	.417	0.60 (0.36, 0.98)	.042	0.69 (0.43, 1.10)		.117
500–1000 euro	0.52 (0.29, 0.93)	.026	1.13 (0.84, 1.52)	.420	1.19 (0.89, 1.60)		.228
< 500 euro	1.18 (0.83, 1.67)	.355	1.36 (0.88, 2.11)	.167	1.29 (0.83, 1.99)		.260
Vaccinated against HPV (yes ref)	–	–	–	–	–		–
No	1.81 (1.38, 2.36)	< .001	2.26 (1.69, 3.02)	< .001	4.19 (3.07, 5.72)		< .001
Against adolescent vaccination (No ref)	–	–	–	–	–		–
Yes	2.21 (1.57, 3.12)	< .001	2.33 (1.65, 3.29)	< .001	3.11 (2.17, 4.44)		< .001

–: indicate refererral categories

### Analysis of knowledge items related to the existence and use of HPV DNA test

Knowledge items related to the existence and utility of the HPV DNA test were less commonly correctly addressed by both males and females that participated in the study (Table 1). Nevertheless, differences among the two groups favored females in terms of correct answers.

Logistic regression analysis revealed male sex, age groups of > 50 and 21–25 years old, serving as a private employee and non-vaccinated status as well as not favoring adolescent vaccination as statistically significant factors that were associated with an increased rate of incorrect responses (Table 2).

### Analysis of knowledge items related to the existence and efficacy of HPV vaccination

Approximately 77.7% of males and 89.8% of females were aware of the existence of an HPV vaccination program in Greece. Only half of men and two out of three women knew that it does not provide full coverage against cervical cancer. Similarly, only one in three men and two in three women, knew that the vaccine should be ideally performed prior to the onset of sexual activity.

Once again, male sex, age group > 50 years old, unemployed status, serving as a private employee or self-employed and being non-vaccinated and not favoring vaccination of adolescents were identified as parameters that were associated with a increased rate of incorrect responses (Table 2). Receiving only basic education also increased the risk of responding incorrectly, whereas age group of participants between 26 and 30 years was associated with a higher chance of correctly identifying most questions.

### Questionnaire validation and evaluation of overall knowledge measure

Rasch analysis was performed separately for males and females, given the differences that were noted in the univariate analysis presented in Table 1. Person reliability index was evaluated as high in both men (reliability 92.2%) and women (85.2%), indicating that both were able to correctly interpret questions and answer them (Appendix). Local independence of included questions was denoted following correlation coefficient analysis of residuals (Appendix Q3 Table).

The median and interquartile number of errors that were identified in the univariate analysis of the 24 questions that were related to the pathophysiology of HPV infection was 8 (5–12). Males had significantly more questions answered incorrectly as opposed to females (11.5 (5–15) vs 8 (5–12)). Similarly, the number of incorrect answers related to the five questions that were posed concerning HPV DNA knowledge was 2 with an IQR that ranged between 1 and 4. Once again males had more questions answered incorrectly compared to females (3 (1–5) vs 2 (1–3)). The median number of errors that were noted in the 9 questions that aimed to measure knowledge on HPV vaccination was 2 with an IQR of 1–4. Similarly, to the previous two categories of questions males were more likely to respond incorrectly compared to females (4 (2–6) vs 2 (1–4)).

The proportion of correctly identified questions per knowledge item is indicated in the Item Statistics Table of Appendix. The difficulty of answering each question has been measured and the provided small standard errors indicate that the precision of the questionnaire in terms of the hierarchy of difficulty is high.

**Fig. 1:**
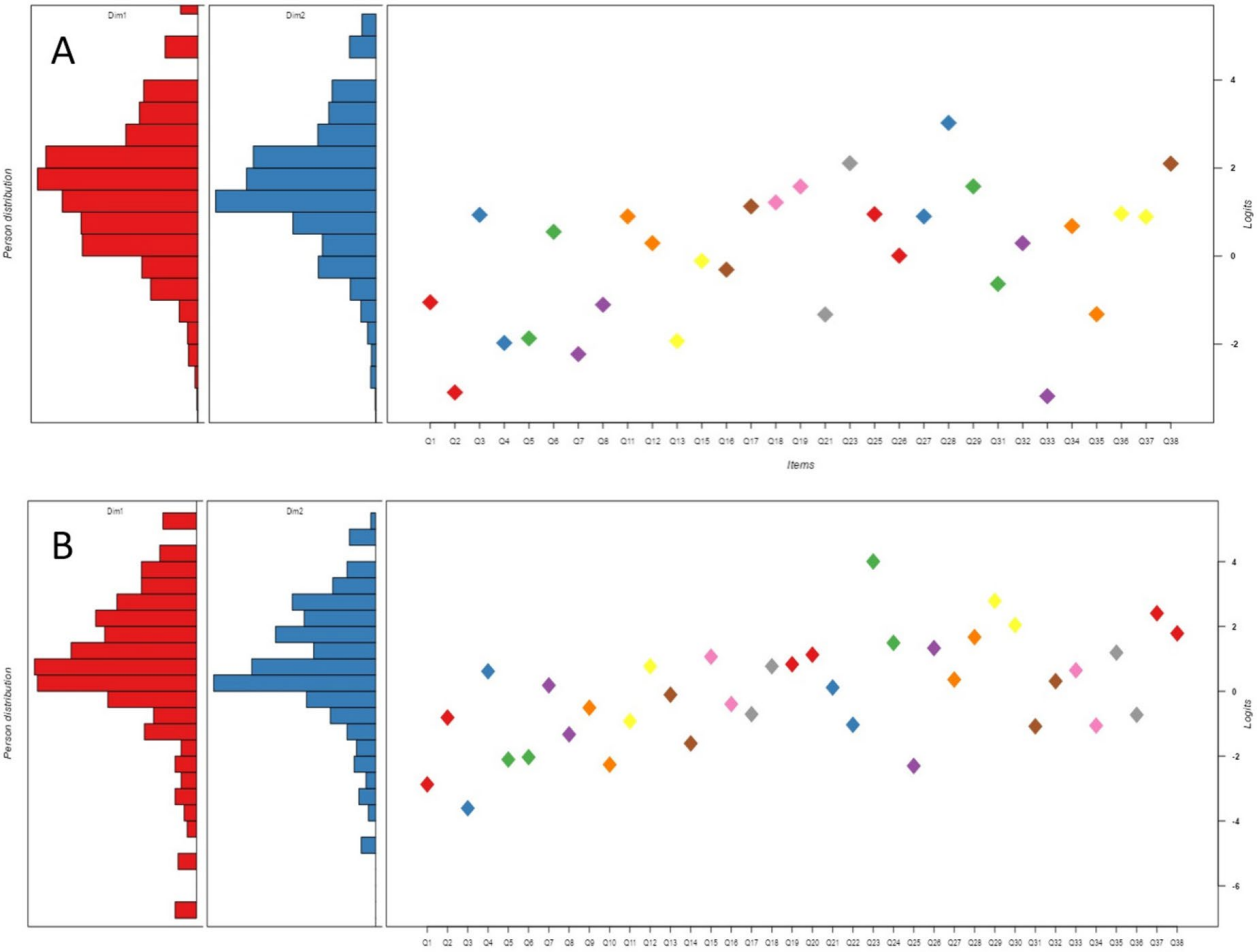
Wright map of the difficulty of responding to questions among women (**A**) and men (**B**) prior (red) and following (blue) the correction of outlier questions (those that were difficult to understand). The logit value of the difficulty of each question is indicated in the scatter dot diagram

Infit and outfit values of Rasch analysis in women indicated that questions No 9,10, 22 and 30 might influence the initial analysis (Appendix Item Statistics and Expected Score Curves). This was not, however, related to their difficulty as indicated in the Wright map (Fig. 1). These questions were removed from the Respondent ability ranged between -4.8 and 6.6 following a normal distribution. Infit and outfit values of Rasch analysis in men indicated that none of the questions contributed to statistical noise (Appendix Item Statistics and Expected Score Curves). Person statistics of men and women (after the exclusion of the previously mentioned questions for women) are presented on Wright maps in Fig. 1.

**Fig. 2:**
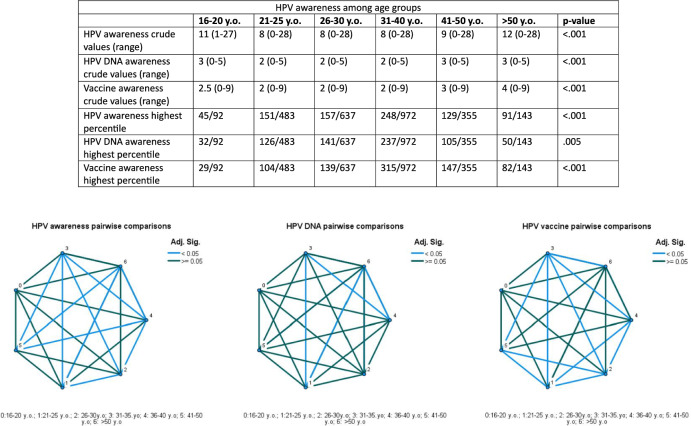
Pairwise comparisons of crude knowledge among the different age groups

Six distinct age groups were collected that included men and women aged 16–20, 21–25, 26–30, 31–35, 36–40, 40–50 and > 50 years old. Participants aged between 26 and 30 years old being constituted the predominant group, followed by those aged 31–35 years (Appendix). Crude differences among the age groups were statistically significant among the included age groups for all three categories including HPV infection, HPV DNA testing and HPV vaccine awareness (*p* < 0.001). Statistically significant differences in pairwise comparisons of crude differences among the 6 identified groups are depicted in Fig. 2. The percentage of participants obtaining the best answers (achieving the highest percentile) also significantly differed among the various groups (*p* < 0.001 for HPV infection; *p* = 0.005 for HPV DNA testing and *p* < 0.001 for HPV vaccination).

## Discussion

### Study findings

The findings of our study reveal that the majority of participants (96.6%) have heard about HPV prior to our survey, only 51.6% of men and 60.4% of women were aware about the high prevalence of HPV infection in general population. It should be noted, however, that male participants were less aware on the pathophysiology of HPV infection compared to female participants and that 9.1% of them weere completely ignorant of the existence of the virus. Similarly, knowledge concerning the availability and utility of HPV vaccination was particularly low among men as only 50% of them knew that HPV immunization does not provide full coverage against cervical cancer and only 33% knew that the vaccination should be ideally performed prior to the onset of sexual activity. Variations were also noted among the different age groups and the most concerning observation was the statistically significantly lower knowledge of HPV awareness among women and men aged 31–50 years compared to the other groups which represent the most important group for the detection of preinvasive and invasive lesions as cervical cancer is mostly encountered in the age group.

### Comparison with existing literature

Similar findings were observed by other researchers in an international level. Specifically, a questionnaire survey which conducted in Japan from 2015 to 2016, among general population and included 3,033 participants of both genders aged over 16 years, concluded that HPV knowledge and awareness, as well as HPV prevention knowledge was of low level between male population [11]. An international web-based survey with 2409 male and female participants, aged 18 to 70 years old, from the USA, UK, and Australia came to the conclusion that only 61% of the participants had heard of HPV, whereas only 50% of them had heard of HPV testing. The researchers also concluded that among those who were aware of HPV testing, women had a higher knowledge score than men [14]. Our results seem more promising, since the great majority of the participants had heard about HPV prior to our survey. In agreement with that study, female participants in our survey were more likely to correctly answer questions about HPV DNA testing and its utility.

To date it remains unknown why men do not possess the necessary knowledge concerning the medical burden of HPV and the necessity of HPV vaccination, however, it is believed that the lack of appropriate education possibly is the result of the absence of pathology among those infected by the virus. However, it should be stressed that the majority of available information is based on questionnaires that address women, whereas data in the male population remain scarce in the international literature [12, 13].

These speculations are supported by a previous study that was based in 298 men that attended the STI clinic and the HIV clinic of Andreas Syggros Hospital in Athens [15]. Researchers observed that 92.6% of the participants identified HPV as a common STI, although only 68% of the correctly identified HPV as a cause of cancer in both sexes, whereas in our survey only half of the participants related HPV with head-, neck- and penile cancer.

Despite the fact that there is no medical link between HPV and HIV, 10–13% of the participants incorrectly believe, that HPV infection may lead to AIDS. Moreover, almost a quarter of participants have the misperception that condom use may provide absolute immunity to HPV. On the contrary, a greek epidemiologic study among 4507 adolescents showed that almost 80% of them knew that the use of condom reduces the risk of HPV infection [16]. This indicates that high HPV awareness rates are not necessary compatible with high knowledge about HPV infection. A Brazilian multicenter survey that included 8581 adults concluded that having high awareness about HPV does not ensure similar levels of knowledge [17].

The high acceptance rate of HPV vaccination which is inferred from the results of our study, opposes the results of the latest systematic review which investigated the HPV knowledge and immunization acceptance among 429,875 European parents. According to that systematic review, only 59.2% of the participants were positive towards vaccination of their children [18]. A study which conducted in Greece from 2005 to 2010 and included 5249 women, to assess the HPV vaccine acceptance rates, interestingly revealed that the acceptance rates dropped after the public availability of HPV vaccine in Greece. In more detail, the acceptance rate for immunization decreased from 81.9 to 65.7% and from 81.6 to 62.6% for the participations’ daughters and sons respectively [19]. Only 15 out of 386 men in our survey reported to be vaccinated, which is explained by the fact that HPV male vaccination was not incorporated in the National Immunization Program in Greece until 2022.

A cross sectional study of 2,002 participants carried out in Australia using a telephone survey concluded that educational programs target groups should include men, young adults and the elderly [20]. These conclusions are complied with our findings, that indicate, that educational interventions about HPV infection and prevention should be targeted in specific subgroups such as males, age groups of > 50 and 21–25 years old, non-vaccinated population and those who are not favoring adolescent vaccination.

### Strengths and limitations

This is the largest online survey among both male and female Greek population, which investigates the existing knowledge and attitudes about prevention, screening, and HPV vaccination. To our knowledge no similar studies have been conducted online in Greece. Wide participation of a diverse population and its timing, which has been extended during the period prior to the onset of immunization of male adolescents, significantly strengthen the importance of our findings. Moreover, the observed reliability of the questionnaire was evaluated as high and observation that is the result of the low complexity of provided questions and answers which were mostly easily answered according to the Rasch, infit and outfit analysis.

On the other hand, certain limitations should be mentioned. Especially, the main weakness of our study is the representativeness of the study population, since 85% of the participants were females. Furthermore, the questionnaire was created with Google forms and distributed through social media, which automatically makes it accessible only to internet users.

## Conclusion

Understanding the level of knowledge and awareness concerning HPV in Greek general population helps policy makers gain an objective opinion about the subcategories that lack of knowledge and organize targeted health promotion and educational programs, to achieve high acceptance rate of vaccination in both sexes and benefit from herd immunity. The findings of this study indicate that although awareness of the existence of HPV infection is high in the general population of Greece perception of the pathophysiology and prevention measures remains limited. Our study also denotes the population categories that need to be targeted to increase knowledge and attitudes towards proper HPV screening that will, evidently, increase the low rates of HPV vaccination.

### Supplementary Information

Below is the link to the electronic supplementary material.

Supplementary file1 (PDF 1155 KB)

## Data Availability

The authors confirm that the data supporting the findings of this study are available within the article and its supplementary materials file.

## References

[1] De Martel C, Ferlay J, Franceschi S, Vignat J, Bray F, Forman D (2012). Global burden of cancers attributable to infections in 2008: A review and synthetic analysis. Lancet Oncol.

[2] Formana D, de Martel C, Lacey CJ, Soerjomataram I, Lortet-Tieulent J, Bruni J (2012). Global burden of human papillomavirus and related diseases. Vaccine.

[3] Anagnostou PA, Aletras VH, Niakas DA (2017). Human papillomavirus knowledge and vaccine acceptability among adolescents in a Greek region. Public Health.

[4] Thanasas I, Lavranos G, Gkogkou P, Paraskevis D (2022). The Effect of Health Education on Adolescents’ Awareness of HPV Infections and Attitudes towards HPV Vaccination in Greece. Int J Environ Res Public Health.

[5] Mammas IN, Theodoridou M, Koutsaftiki C, Bertsias G, Sourvinos G, Spandidos DA (2016). Vaccination against Human Papillomavirus in relation to Financial Crisis: The “Evaluation and Education of Greek Female Adolescents on Human Papillomaviruses’’ Prevention Strategies" ELEFTHERIA Study”. J Pediatr Adolesc Gynecol.

[6] Valasoulis G, Pouliakis A, Michail G, Daponte AI, Galazios G, Panayiotides IG (2021). The influence of sexual behavior and demographic characteristics in the expression of HPV-related biomarkers in a colposcopy population of reproductive age greek women. Biology (Basel)..

[7] Gunasekaran B, Jayasinghe Y, Fenner Y, Moore EE, Wark JD, Fletcher A (2012). Knowledge of human papillomavirus and cervical cancer among young women recruited using a social networking site. Sex Transm Infect.

[8] Miyagi E, Motoki Y, AsaiSato M, Taguri M, Morita S, Hirahara E (2014). Web-based recruiting for a survey on knowledge and awareness of cervical cancer prevention among young women living in Kanagawa Prefecture. Japan. Int J Gynecol Cancer..

[9] Smyth JD, Dillman DA, Christian LM, Stern MJ (2006). Comparing check-all and forced-choice question formats in Web surveys. Public Opin Q.

[10] Callegaro M, Murakami M, Tepman Z, Henderson V (2015). Yes–no answers versus check-all in self-administered modes: a systematic review and analyses. Int J Mark Res.

[11] Suzuki Y, Sukegawa A, Nishikawa A, Kubota K, Motoki Y, Asai-Sato M (2019). Current knowledge of and attitudes toward human papillomavirus-related disease prevention among Japanese: A large-scale questionnaire study. J Obstet Gynaecol Res.

[12] Durusoy R, Yamazhan M, Taşbakan MI, Ergin I, Aysin M, Pullukçu H (2010). HPV vaccine awareness and willingness of first-year students entering university in Western Turkey. Asian Pac J Cancer Prev.

[13] Horvath JDC, Kops NL, Caierão J, Bessel M, Hohenberger G, Wendland EM (2018). Human papillomavirus knowledge, beliefs, and behaviors: A questionnaire adaptation. Eur J Obstet Gynecol Reprod Biol.

[14] Dodd RH, McCaffery KJ, Marlow LA, Ostini R, Zimet GD, Waller J (2014). Knowledge of human papillomavirus (HPV) testing in the USA, the UK and Australia: an international survey. Sex Transm Infect.

[15] Hoefer L, Tsikis S, Bethimoutis G, Nicolaidou E, Paparizos V, Antoniou C (2018). HPV vaccine acceptability in high-risk Greek men. Hum Vaccin Immunother.

[16] Vaidakis D, Moustaki I, Zervas I, Barbouni A, Merakou K, Chrysi MS (2017). Knowledge of Greek adolescents on human papilloma virus (HPV) and vaccination: A national epidemiologic study. Medicine (Baltimore).

[17] Kops NL, Hohenberger GF, Bessel M, Correia Horvath JD, Domingues C (2019). Knowledge about HPV and vaccination among young adult men and women: Results of a national survey. Papillomavirus Res.

[18] López N, Garcés-Sánchez M, Panizo MB, de la Cueva IS, Artés MT, Ramos Bópez N, Garcés-Sánchez M (2020). HPV knowledge and vaccine acceptance among European adolescents and their parents: a systematic literature review [published correction appears in Public Health Rev. 2020 Jul 21;41:20]. Public Health Rev.

[19] Sotiriadis A, Dagklis T, Siamanta V, Chatzigeorgiou K, Agorastos T (2012). Increasing fear of adverse effects drops intention to vaccinate after the introduction of prophylactic HPV vaccine. Arch Gynecol Obstet.

[20] Marshall H, Ryan P, Roberton D, Baghurst P (2007). A cross-sectional survey to assess community attitudes to introduction of Human papillomavirus vaccine. Aust N Z J Public Health.

