# Supplementary material for: Awareness, knowledge and attitudes of human papillomavirus infection, screening and vaccination: a survey study in Greece
Source: Arch Gynecol Obstet. 2024 Mar 8;309(5):2031–40. doi: 10.1007/s00404-024-07398-1 (PMC11018653; doi:10.1007/s00404-024-07398-1)
Supplement: Supplementary file 1 — Supplementary file1 (PDF 1155 KB) [file 404_2024_7398_MOESM1_ESM.pdf]

Awareness, Knowledge and Attitudes of Human Papillomavirus Infection, Screening and Vaccination: A Survey Study in Greece.

Human Papillomavirus (HPV) and Vaccination. The human papillomavirus (HPV) is a group of viruses that are particularly common worldwide. The aim of vaccination against HPV is to limit the cases that may develop precancerous lesions and, by extension, cervical cancer. The purpose of this study is to outline the level of public awareness regarding the infection caused by the Human Papillomavirus (HPV) as well as the level of sensitivity regarding vaccination against HPV. The questionnaire is anonymous, and none of the study's creators is aware of the participants' identities. It takes approximately 5 minutes to complete.

I wish to participate in the study:

Yes

No

Demographic Information

In this section, you are invited to respond to basic questions regarding your demographic information. You have the option to decline to answer.

1. Gender: *

Male

Female

I prefer not to answer this question.

2. Nationality:

(Your answer)

3. Age: *

16-20

21-25

26-30

31-35

36-40

41-45

46-50

51 and above

I prefer not to answer this question.

4. Education Level: *

Primary (up to High School)

Vocational Training Institute (IEK)

Technological Educational Institute (TEI)

University (AEI)

Polytechnic University

Holder of a Master's degree

Holder of a Doctorate

I prefer not to answer this question.

5. Marital Status: *

Married

Single

Divorced

I prefer not to answer this question.

6. Have you had a sexual intercourse? *

Yes

No

I prefer not to answer this question.

7. Number of Children: *

None

1

2

3

4

5

6 or more

I prefer not to answer this question.

8. Occupational Status: *

Employed

Unemployed

University student

Pupil

I prefer not to answer this question.

9. If employed, select the professional sector you belong to: *

Private Sector

Public Sector

Self-employed

I prefer not to answer this question.

10. What is your monthly individual income? *

<500 euros

500 - 1000 euros

1000 - 1500 euros

1500 - 2000 euros

>2000 euros

I prefer not to answer this question

Current Knowledge Regarding the Papillomavirus

In this section, you are asked to answer questions related to the infection caused by the human papillomavirus (HPV).

1. Had you heard about the Human Papillomavirus (HPV) before participating in this study? *

Yes

No

2. Are there many different types of HPV viruses? *

Correct

Incorrect

I don't know.

3. Can HPV be transmitted through sexual contact? *

Correct

Incorrect

I don't know.

4. Is HPV transmitted exclusively through sexual contact? *

Correct

Incorrect

I don't know

5. Only women can be infected by HPV viruses. *

Correct

Incorrect

I don't know

6. Men cannot be infected by HPV viruses. *

Correct

Incorrect

I don't know.

7. The use of condoms provides complete protection against HPV viruses. *

Correct

Incorrect

I don't know.

8. Infection by HPV is a rare occurrence. *

Correct

Incorrect

I don't know.

9. Infection by HPV always causes visible signs and symptoms. *

Correct

Incorrect

I don't know.

10. Having multiple sexual partners increases the risk of contracting HPV. *

Correct

Incorrect

I don't know.

11. Someone without symptoms cannot transmit HPV. *

Correct

Incorrect

I don't know.

12. Most sexually active men and women will be infected by HPV at some point in their lives. *

Correct

Incorrect

I don't know.

13. HPV infection... *

Causes HIV/AIDS.

Can cause cervical cancer.

Is treated with antibiotics.

Can be transmitted through oral sex.

Can be transmitted through anal sex.

Can cause oropharyngeal cancer.

Can cause rectal cancer.

Can cause penile cancer.

Can cause herpes.

Can cause warts.

Usually does not require any treatment.

Can cause growths in the airway (respiratory papillomatosis).

14. Do you know what cervical cancer is? *

Yes

No

15. With the HPV test, we detect oncogenic types of HPV directly in the cervical cells. *

Correct

Incorrect

I don't know.

16. If a woman tests positive for HPV, she will definitely develop cervical cancer. *

Correct

Incorrect

I don't know.

17. The HPV test is used to determine if vaccination against HPV is required. *

Correct

Incorrect

I don't know.

18. The PAP test detects HPV viruses in women. *

Correct

Incorrect

I don't know.

19. The HPV test can be performed at the same time with the PAP test. *

Correct

Incorrect

I don't know.

Current Knowledge About HPV Vaccination

In this section, you are asked to answer questions related to the availability and dynamics of the Human Papillomavirus (HPV) vaccine.

1. Is there a vaccine that protects against HPV infection? *

Yes

No

I don't know.

2. The HPV vaccine is suitable only for individuals with an active sex life. *

Correct

Incorrect

I don't know.

3. Any woman who has been vaccinated against HPV will not develop cervical cancer. *

Correct

Incorrect

I don't know.

4. Women who are vaccinated against HPV do not need to undergo Pap tests again. *

Correct

Incorrect

I don't know.

5. HPV vaccines provide protection against all types of HPV. *

Correct

Incorrect

I don't know.

6. HPV vaccines provide protection against all sexually transmitted infections. *

Correct

Incorrect

I don't know.

7. HPV vaccination requires the administration of only one vaccine dose. *

Correct

Incorrect

I don't know.

8. HPV vaccines are more effective when administered to individuals who have not had sexual intercourse in the past. *

Correct

Incorrect

I don't know.

9. There are HPV vaccines that offer protection against genital warts. *

Correct

Incorrect

I don't know.

10. How many different HPV vaccines are available? *

1

2

3

4

5

More than 5

I don't know.

11. Have you been vaccinated against HPV? *

Yes

No

12. Do you believe that adolescents should be vaccinated against HPV? *

Yes

No

Table Appendix. Baseline characteristics of participants	
	**N (%)**
**Gender**	
• Male	386 (14.4%)
• Female	2,285 (85.1%)
• No response	14 (0.5%)
**Ethnicity**	
• Greek	2,326 (86.6%)
• Other	32 (1.2%)
• No response	327 (12.2%)
**Age Groups (years)**	
• 16 – 20	92 (3.4%)
• 21 – 25	483 (18%)
• 26 – 30	637 (23.7%)
• 31 – 35	556 (20.7%)
• 36 – 40	416 (15.5%)
• 41 – 45	244 (9.1%)
• 46 – 50	111 (4.1%)
• ≥ 51	143 (5.3%)
• No response	3 (0.1%)
**Educational Level**	
• Primary and/or Secondary Education	249 (9.3%)
• Bachelor's or equivalent level	1,673 (62.3%)
• Master's or equivalent level	685 (25.5%)
• Doctoral or equivalent level	58 (2.2%)
• No response	20 (0.7%)
**Marital Status**	
• Married	927 (34.5%)
• Single	1,605 (59.8%)
• Divorced	117 (4.4%)
• No response	36 (1.3%)
**Parental Status**	
• No children	1,806 (67.3%)
• 1	385 (14.3%)
• 2	367 (13.7%)
• 3	92 (3.4%)

• ≥ 4	22 (0.8%)
• No response	13 (0.5%)
**Employment Status**	
• Employed	2,027 (75.5%)
• Unemployed	256 (9.5%)
• School student	16 (0.6%)
• University student	344 (12.8%)
• No response	42 (1.6%)
**Sexual activity**	
• Started	2,574 (95.9%)
• Non-started	95 (3.5%)
• No response	16 (0.6%)
**Monthly Income (euro)**	
• < 500 euro	496 (18.5%)
• 500 – 1,000 euro	987 (36.8%)
• 1,000 – 1,500 euro	517 (19.3%)
• 1,500 – 2,000 euro	181 (6.7%)
• > 2,000 euro	149 (5.5%)
• No response	355 (13.2%)
**Working position**	
• Private employee	1,252 (46.6%)
• Public employee	598 (22.3%)
• Independent employee	314 (11.7%)
• No response	521 (19.4%)
**Vaccination Status (against HPV)**	
• Vaccinated	932 (34.7%)
• Non-vaccinated	1,753 (65.3%)

Rasch model (men)

Results

Dichotomous Rasch Model

Each variable must be **coded as 0 or 1 with the type of numeric-continuous** in jamovi.

The results of **Save** will be displayed in the datasheet.

The result tables are estimated by Marginal Maximum Likelihood estimation(MMLE).

The rationale of snowIRT module is described in the documentation.

Feature requests and bug reports can be made on my GitHub.

Model Fit

	Person Reliability
scale	0.922

[3]

Q3 Correlation Matrix

	Q1	Q2	Q3	Q4	Q5	Q6	Q7	Q8	Q9	Q10	Q11	Q12	Q13	Q14	Q15	Q16	Q17	Q18	Q19	Q
Q1	—																			
Q2	0.053	—																		
Q3	0.173	-0.002	—																	
Q4	0.006	0.065	0.039	—																
Q5	0.029	-0.009	0.216	0.083	—															
Q6	0.046	-0.030	0.165	0.093	0.689	—														
Q7	0.013	0.031	-0.008	0.110	0.108	0.123	—													
Q8	0.055	0.027	0.001	0.028	0.129	0.098	0.056	—												
Q9	0.066	0.011	0.007	0.124	0.046	0.079	0.132	0.240	—											
Q10	-0.071	-0.044	0.080	-0.092	0.092	0.025	0.001	0.023	0.014	—										
Q11	-0.155	-0.066	0.001	0.015	0.050	-0.029	-0.154	0.139	0.196	0.004	—									
Q12	0.010	-0.014	0.011	-0.126	-0.073	-0.032	0.030	0.146	0.091	0.100	-0.040	—								
Q13	0.135	0.006	0.045	0.083	-0.100	-0.093	0.130	0.038	0.165	-0.036	-0.208	0.074	—							
Q14	0.126	-0.081	0.033	-0.112	-0.214	-0.147	-0.142	-0.166	-0.110	-0.060	-0.030	-0.035	0.035	—						
Q15	-0.042	-0.087	-0.038	0.060	-0.089	0.002	-0.004	-0.048	-0.069	-0.110	-0.106	-0.022	0.065	0.063	—					
Q16	-0.053	-0.031	-0.004	0.013	0.112	0.138	0.041	0.096	-0.008	0.135	-0.046	0.029	0.003	-0.015	-0.068	—				
Q17	0.002	0.118	0.030	-0.011	0.166	0.103	0.071	0.028	-0.082	0.208	-0.044	0.051	-0.025	-0.022	-0.173	0.401	—			
Q18	-0.070	-0.112	-0.064	-0.168	-0.098	0.001	-0.126	-0.121	-0.079	0.022	-0.017	-0.030	-0.144	0.094	-0.121	0.250	0.058	—		
Q19	-0.046	0.005	-0.030	-0.152	0.009	-0.038	-0.048	-0.093	-0.170	-0.014	-0.020	-0.064	-0.236	0.081	-0.112	0.087	0.252	0.512	—	
Q20	-0.077	0.051	-0.066	-0.116	-0.081	0.008	-0.044	-0.104	-0.166	-0.075	-0.031	-0.104	-0.243	0.083	-0.028	0.019	-0.005	0.422	0.529	
Q21	-0.038	-0.038	-0.093	-0.094	-0.111	-0.155	-0.100	-0.125	-0.165	0.047	-0.020	-0.036	-0.122	0.073	0.006	-0.090	-0.051	0.070	0.099	0.
Q22	0.021	-0.050	-0.012	-0.095	-0.032	-0.051	0.027	0.043	0.015	-0.074	-0.094	0.024	0.027	0.029	-0.055	-0.025	-0.074	0.062	0.107	0.
Q23	-0.055	0.039	-0.015	-0.033	0.005	0.010	-0.056	0.003	-0.098	0.014	0.004	-0.006	-0.060	-0.039	-0.052	-0.063	-0.030	-0.068	-0.045	-0.
Q24	-0.015	0.004	-0.049	-0.004	-0.068	-0.065	0.055	0.018	-0.082	-0.068	-0.076	-0.036	-0.075	-0.043	0.003	-0.015	0.018	0.092	0.119	0.
Q25	-0.007	0.092	-0.023	-0.027	-0.161	-0.165	-0.116	-0.021	-0.064	-0.000	-0.014	-0.034	-0.029	0.055	0.005	-0.155	-0.046	-0.022	0.011	-0.
Q26	-0.044	-0.049	-0.050	-0.219	-0.097	-0.063	-0.156	-0.130	-0.181	0.014	-0.023	-0.050	-0.170	0.042	0.020	-0.163	-0.144	-0.034	-0.104	0.
Q27	-0.070	0.040	-0.085	-0.104	0.009	-0.001	-0.025	0.081	0.169	-0.120	0.024	-0.063	0.038	0.025	-0.069	-0.088	-0.163	-0.116	-0.203	-0.
Q28	-0.097	-0.090	0.007	-0.019	0.005	0.026	-0.114	-0.104	-0.075	-0.079	0.057	-0.080	-0.097	-0.041	-0.023	-0.131	-0.127	-0.060	-0.119	-0.
Q29	-0.035	-0.027	-0.124	-0.105	-0.061	-0.017	-0.127	-0.001	-0.040	-0.062	0.029	-0.065	-0.003	-0.091	0.072	-0.065	-0.134	-0.167	-0.190	-0.
Q30	-0.007	-0.007	0.032	-0.131	-0.031	-0.064	-0.039	-0.058	-0.055	0.032	0.019	-0.026	-0.114	-0.005	-0.035	-0.107	-0.078	-0.091	-0.072	-0.
Q31	0.054	-0.043	-0.055	-0.009	-0.130	-0.136	-0.146	-0.235	-0.224	0.001	-0.165	-0.077	0.051	0.067	0.039	-0.033	-0.144	-0.072	-0.113	-0.
Q32	-0.069	-0.094	-0.002	0.015	-0.081	-0.106	-0.026	-0.105	-0.120	-0.071	-0.034	-0.139	-0.040	-0.009	-0.051	-0.083	-0.108	-0.146	-0.124	-0.
Q33	-0.057	-0.049	-0.065	0.008	-0.068	-0.127	-0.054	-0.094	0.009	-0.135	0.034	-0.176	0.036	-0.075	-0.020	-0.197	-0.147	-0.208	-0.141	-0.
Q34	-0.052	-0.141	-0.011	-0.106	-0.027	-0.080	-0.110	-0.037	0.023	-0.103	0.052	-0.064	-0.093	0.007	-0.023	-0.039	-0.162	-0.100	-0.124	-0.
Q35	-0.102	0.017	-0.083	0.133	-0.027	-0.052	-0.027	-0.060	-0.026	-0.081	-0.007	-0.131	-0.104	-0.098	-0.059	-0.132	-0.130	-0.027	-0.017	-0.
Q36	0.063	-0.050	-0.017	0.047	-0.010	-0.124	0.013	-0.013	0.060	-0.102	0.008	-0.068	0.128	-0.009	-0.110	-0.155	-0.076	-0.195	-0.123	-0.
Q37	-0.014	-0.079	-0.013	-0.083	-0.101	-0.080	-0.110	-0.054	-0.156	-0.036	-0.111	-0.050	0.017	-0.021	0.059	-0.156	-0.096	-0.054	-0.128	-0.
Q38	0.023	-0.120	-0.041	-0.142	-0.049	-0.076	-0.139	-0.056	-0.130	0.067	-0.105	0.013	-0.030	-0.009	0.051	-0.049	-0.032	-0.019	-0.100	-0.

[3]

Item Statistics

	Proportion	Measure	S.E.Measure	Infit	Outfit
Q1	0.907	-3.4617	0.216	0.907	0.606
Q2	0.748	-1.5791	0.143	0.917	0.847
Q3	0.935	-4.0506	0.249	0.816	2.692
Q4	0.545	-0.1874	0.124	1.081	1.130
Q5	0.865	-2.7913	0.184	0.738	0.571
Q6	0.860	-2.7247	0.181	0.812	0.653
Q7	0.613	-0.6114	0.127	1.017	1.038
Q8	0.802	-2.0735	0.157	0.782	0.607
Q9	0.710	-1.2846	0.137	0.926	0.707
Q10	0.875	-2.9311	0.190	0.925	1.120
Q11	0.760	-1.6838	0.146	1.074	1.298
Q12	0.520	-0.0342	0.124	1.094	1.070
Q13	0.655	-0.8927	0.130	1.023	0.985
Q14	0.828	-2.3345	0.166	0.918	0.637
Q15	0.472	0.2550	0.123	0.983	0.985
Q16	0.695	-1.1738	0.135	0.930	0.716
Q17	0.735	-1.4778	0.141	0.947	0.882
Q18	0.520	-0.0342	0.124	0.896	0.747
Q19	0.510	0.0268	0.123	0.856	0.728
Q20	0.463	0.3160	0.124	0.954	0.873
Q21	0.623	-0.6763	0.128	1.117	1.317
Q22	0.772	-1.7927	0.149	0.868	0.663
Q23	0.100	3.2340	0.190	1.268	1.992
Q24	0.405	0.6705	0.125	0.951	0.857
Q25	0.877	-2.9675	0.192	1.429	1.407
Q26	0.430	0.5153	0.124	1.253	1.502
Q27	0.585	-0.4361	0.126	0.894	0.712
Q28	0.378	0.8441	0.126	1.192	1.241
Q29	0.225	1.9260	0.143	1.088	2.132
Q30	0.323	1.2054	0.130	1.049	1.280
Q31	0.777	-1.8374	0.150	1.183	1.391
Q32	0.593	-0.4835	0.126	1.075	1.197
Q33	0.540	-0.1566	0.124	0.997	1.017
Q34	0.775	-1.8150	0.150	0.868	0.661
Q35	0.453	0.3771	0.124	0.808	0.695
Q36	0.738	-1.4978	0.142	0.916	0.886
Q37	0.273	1.5580	0.136	1.050	1.375
Q38	0.360	0.9568	0.127	1.079	1.020

Note. Infit= Information-weighted mean square statistic; Outfit= Outlier-sensitive means square statistic.

[3]

Expected Score Curve

Q1

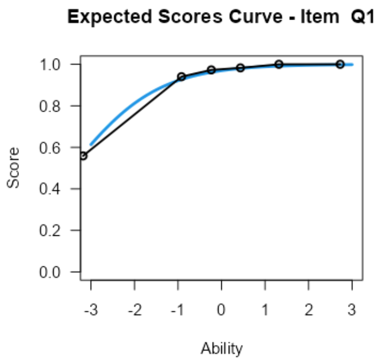

Q2

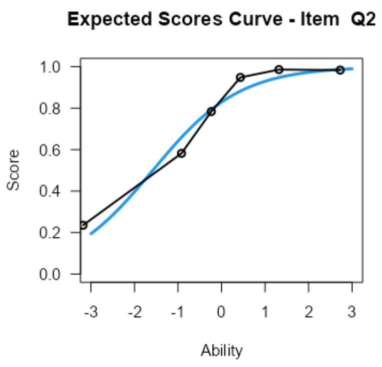
Expected Scores Curve - Item Q2
Score
Ability
1.0
0.8
0.6
0.4
0.2
0.0
-3
-2
-1
0
1
2
3

Q3

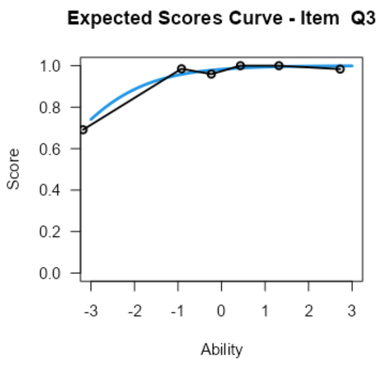
Expected Scores Curve - Item Q3
Score
Ability
1.0
0.8
0.6
0.4
0.2
0.0
-3
-2
-1
0
1
2
3

Q4

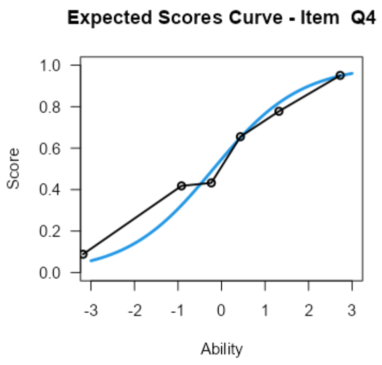
Expected Scores Curve - Item Q4
Score
Ability
1.0
0.8
0.6
0.4
0.2
0.0
-3
-2
-1
0
1
2
3

Q5

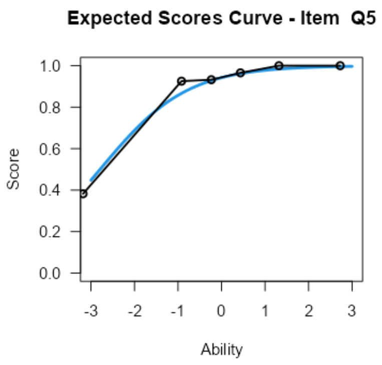
Expected Scores Curve - Item Q5
Score
Ability
1.0
0.8
0.6
0.4
0.2
0.0
-3
-2
-1
0
1
2
3

Q6

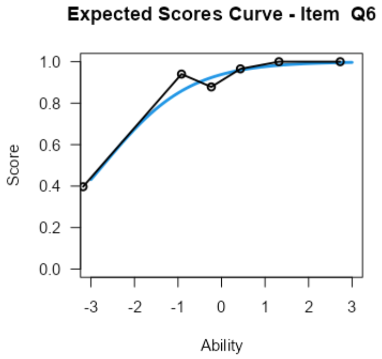
Expected Scores Curve - Item Q6
Score
1.0
0.8
0.6
0.4
0.2
0.0
-3
-2
-1
0
1
2
3
Ability

Q7

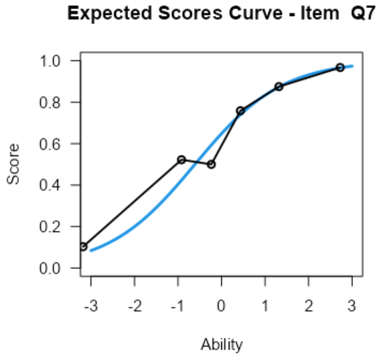
Expected Scores Curve - Item Q7
Score
1.0
0.8
0.6
0.4
0.2
0.0
-3
-2
-1
0
1
2
3
Ability

Q8

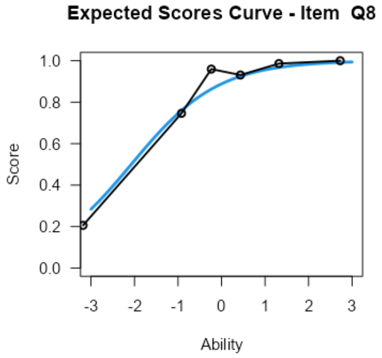
Expected Scores Curve - Item Q8
Score
1.0
0.8
0.6
0.4
0.2
0.0
-3
-2
-1
0
1
2
3
Ability

Q9

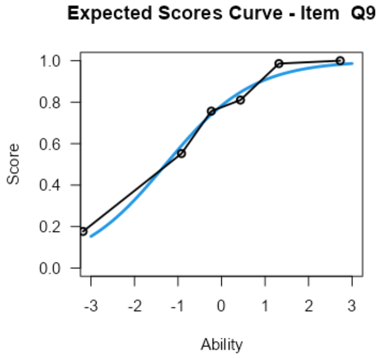
Expected Scores Curve - Item Q9
Score
1.0
0.8
0.6
0.4
0.2
0.0
-3
-2
-1
0
1
2
3
Ability

Q10

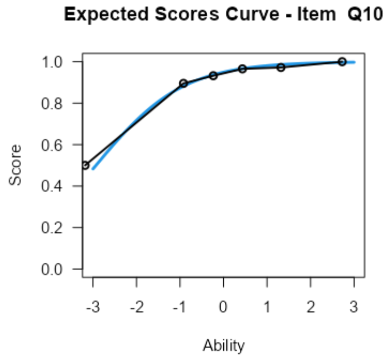

Q11

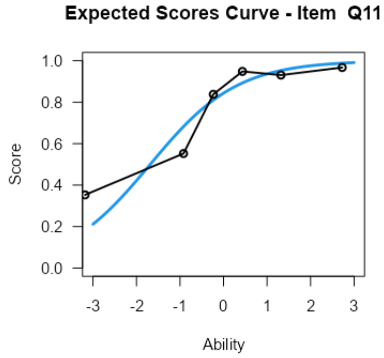

Q12

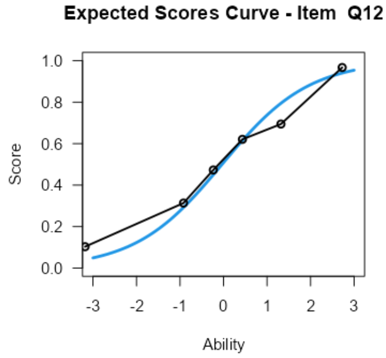

Q13

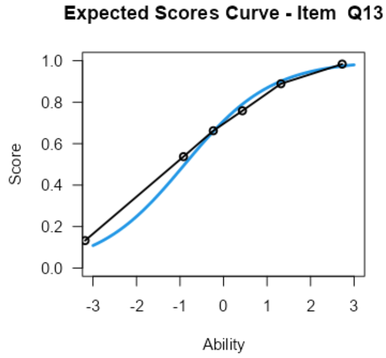

Q14

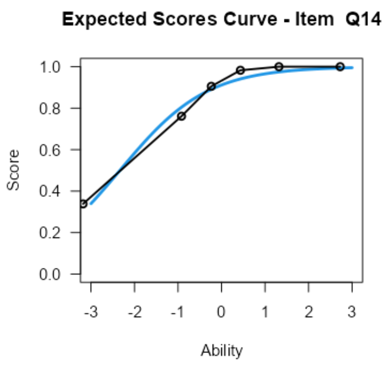

Q15

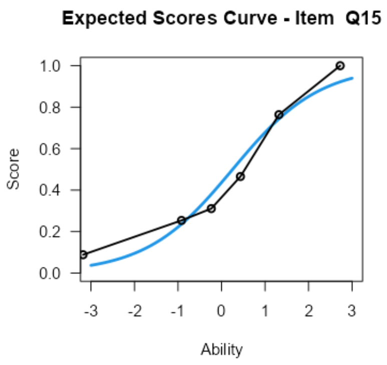

Q16

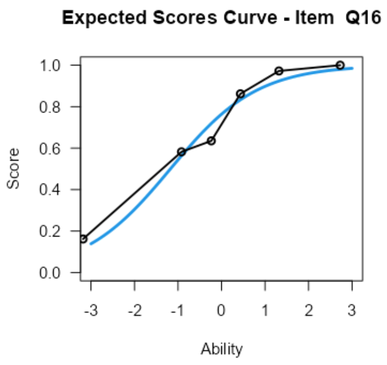

Q17

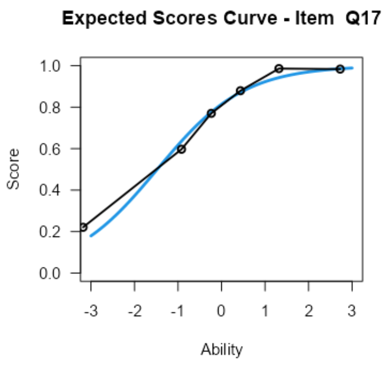

Q18

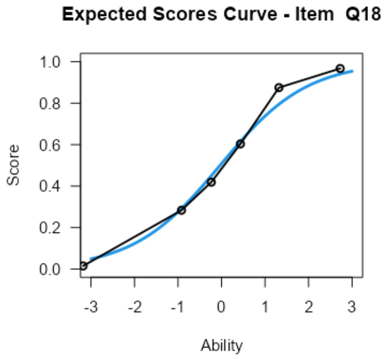

Q19

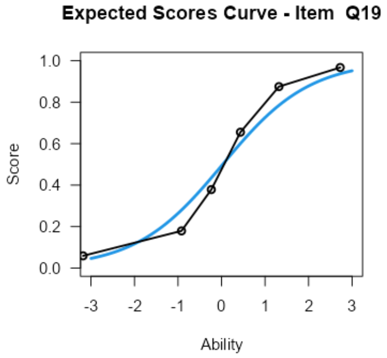

Q20

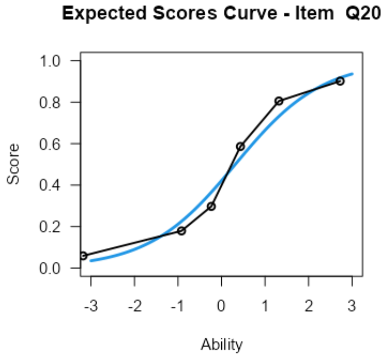

Q21

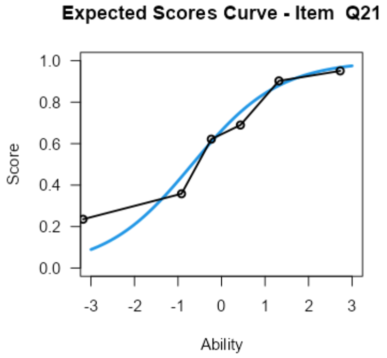

Q22

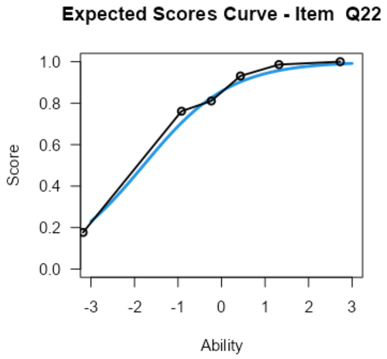

Q23

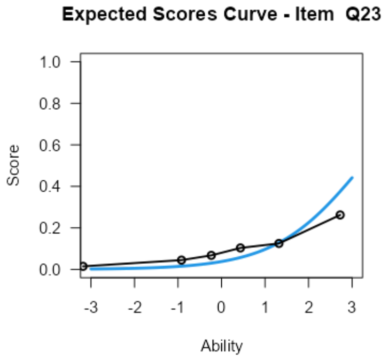

Q24

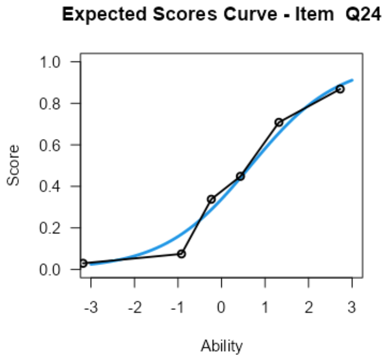

Q25

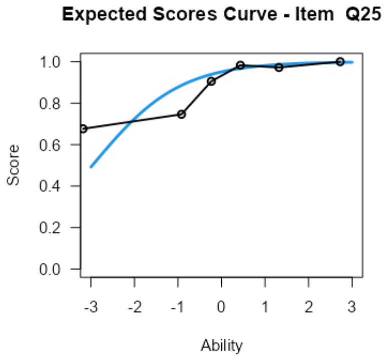

Q26

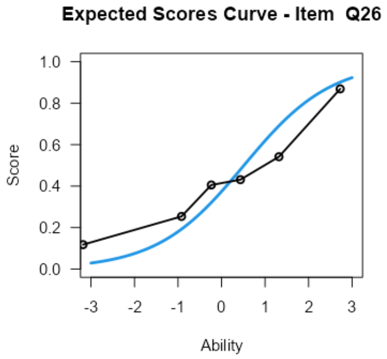

Q27

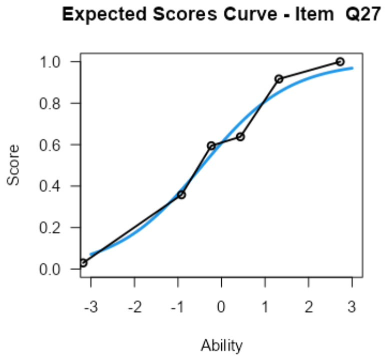

Q28

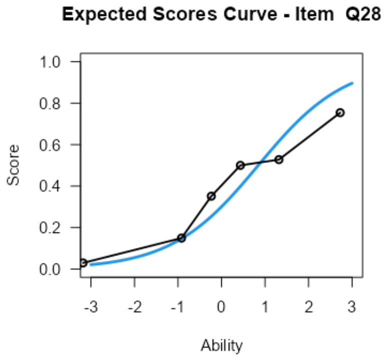

Q29

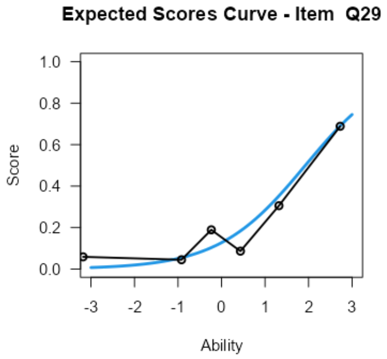

Q30

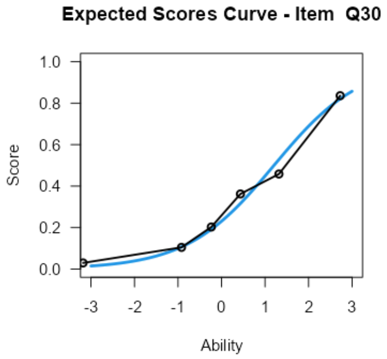

Q31

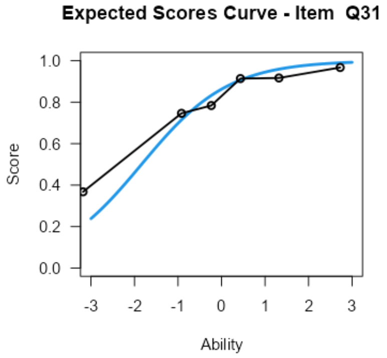

Q32

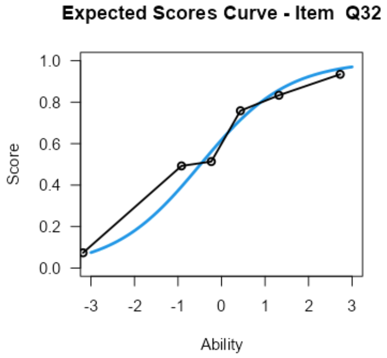

Q33

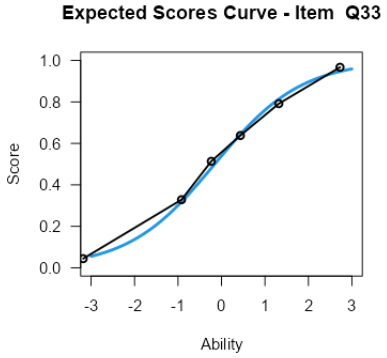

Q34

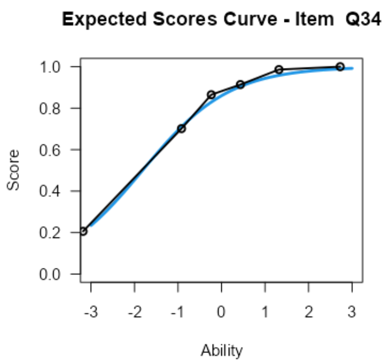

Q35

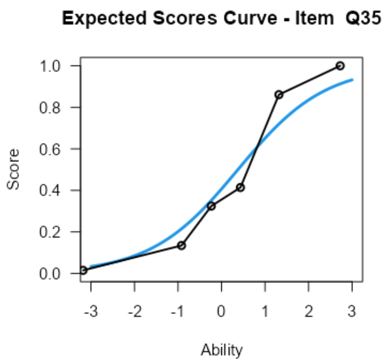

Q36

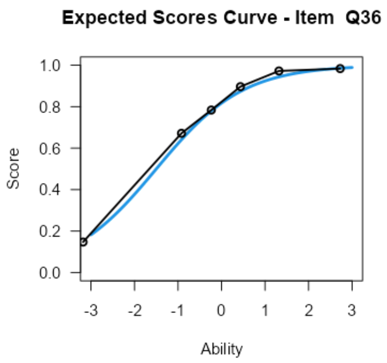

Q37

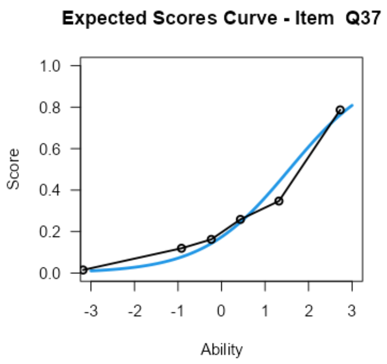

Q38

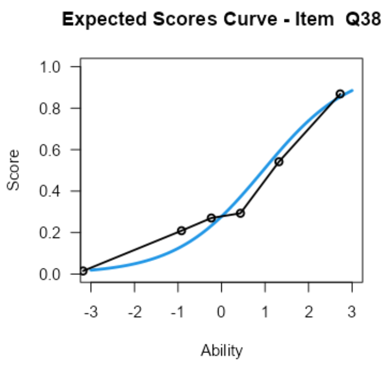

References

[1] The jamovi project (2022). *jamovi*. (Version 2.3) [Computer Software]. Retrieved from https://www.jamovi.org.

[2] R Core Team (2021). *R: A Language and environment for statistical computing*. (Version 4.1) [Computer software]. Retrieved from https://cran.r-project.org. (R packages retrieved from MRAN snapshot 2022-01-01).

[3] Robitzsch,A., Kiefer, T., & Wu, M. (2020). *TAM: Test Analysis Modules*. [R package]. Retrieved from https://CRAN.R-project.org/package=TAM.

[4] Seol, H. (2020). *snowIRT: Item Response Theory for jamovi*. [jamovi module]. Retrieved from https://github.com/hyunsooseol/snowIRT.

Rasch model women

Results

Dichotomous Rasch Model

Each variable must be **coded as 0 or 1 with the type of numeric-continuous** in jamovi.

The results of **Save** will be displayed in the datasheet.

The result tables are estimated by Marginal Maximum Likelihood estimation(MMLE).

The rationale of snowIRT module is described in the documentation.

Feature requests and bug reports can be made on my GitHub.

Model Fit

	Person Reliability
scale	0.852

[3]

Q3 Correlation Matrix

	Q1	Q2	Q3	Q4	Q5	Q6	Q7	Q8	Q9	Q10	Q11	Q12	Q13	Q15	Q16	Q17	Q18	Q19	Q21	Q
Q1	—																			
Q2	0.040	—																		
Q3	-0.033	0.010	—																	
Q4	0.053	0.192	0.016	—																
Q5	0.041	0.188	0.003	0.890	—															
Q6	-0.010	-0.017	0.111	0.020	0.022	—														
Q7	0.034	0.091	-0.012	0.043	0.031	-0.009	—													
Q8	-0.007	0.074	0.008	0.087	0.088	0.077	0.095	—												
Q9	-0.068	0.069	-0.077	-0.062	-0.063	-0.069	0.020	-0.072	—											
Q10	-0.029	0.053	-0.024	0.009	0.018	-0.067	-0.012	0.102	0.077	—										
Q11	0.042	0.020	-0.104	0.017	0.009	0.040	0.087	-0.000	0.001	-0.023	—									
Q12	-0.008	-0.046	-0.080	-0.022	-0.022	0.023	0.007	0.066	-0.065	-0.016	0.095	—								
Q13	0.057	-0.065	-0.128	-0.130	-0.118	-0.034	-0.001	-0.046	-0.021	-0.062	0.042	0.053	—							
Q15	0.015	0.067	0.076	0.058	0.058	0.035	-0.013	0.013	-0.068	-0.055	-0.054	-0.107	-0.129	—						
Q16	0.017	0.058	0.025	0.089	0.076	0.027	-0.010	-0.005	-0.062	-0.027	-0.034	-0.090	-0.076	0.381	—					
Q17	-0.015	-0.009	-0.075	-0.020	-0.035	-0.093	-0.028	-0.073	0.012	-0.071	-0.061	-0.141	0.057	0.267	0.104	—				
Q18	-0.004	-0.008	-0.097	-0.022	-0.043	-0.092	-0.050	-0.088	-0.022	-0.057	-0.059	-0.162	0.064	0.119	0.266	0.530	—			
Q19	-0.029	-0.017	-0.071	-0.009	-0.017	-0.097	-0.058	-0.080	0.008	-0.017	-0.114	-0.208	0.059	0.066	0.109	0.340	0.551	—		
Q21	-0.008	0.120	-0.058	0.014	0.023	-0.052	0.040	0.021	0.032	-0.006	0.022	-0.017	0.128	-0.023	-0.014	0.064	0.011	0.013	—	
Q22	0.015	-0.007	-0.055	-0.015	-0.023	-0.030	0.005	-0.017	-0.045	-0.011	0.075	0.013	-0.006	-0.043	-0.045	-0.077	-0.106	-0.113	-0.036	
Q23	-0.006	-0.030	0.005	-0.029	-0.022	-0.055	-0.009	-0.009	-0.019	-0.039	-0.095	-0.134	-0.030	0.055	0.034	0.155	0.106	0.121	0.011	-0.
Q25	0.013	-0.083	-0.122	-0.120	-0.128	-0.138	-0.008	-0.089	0.034	-0.059	0.027	-0.040	0.005	-0.136	-0.110	-0.099	-0.091	-0.033	-0.021	-0.
Q26	0.029	-0.003	0.004	-0.032	-0.024	0.021	-0.006	0.105	-0.079	-0.008	-0.020	0.102	-0.030	-0.106	-0.119	-0.192	-0.177	-0.164	-0.020	0.
Q27	-0.060	-0.059	-0.046	-0.088	-0.086	-0.070	-0.015	-0.032	-0.050	-0.069	-0.076	0.007	-0.022	-0.110	-0.110	-0.161	-0.142	-0.148	-0.141	0.
Q28	-0.026	-0.003	-0.049	-0.040	-0.042	-0.016	-0.001	-0.018	-0.005	-0.018	-0.047	0.031	-0.034	-0.056	-0.047	-0.065	-0.083	-0.102	-0.046	0.
Q29	-0.011	-0.029	-0.111	-0.090	-0.088	-0.137	-0.041	-0.060	-0.000	-0.028	-0.016	-0.009	-0.042	-0.133	-0.120	-0.090	-0.111	-0.067	-0.038	-0.
Q30	-0.024	-0.076	-0.039	-0.087	-0.071	-0.098	-0.079	-0.044	-0.091	-0.085	-0.066	0.042	0.093	-0.118	-0.082	-0.046	-0.082	-0.062	-0.064	-0.
Q31	-0.108	-0.096	0.018	-0.127	-0.117	-0.102	-0.022	-0.006	-0.099	-0.017	-0.076	0.008	0.068	-0.112	-0.128	-0.074	-0.094	-0.091	-0.061	-0.
Q32	-0.064	-0.072	-0.021	-0.044	-0.026	0.032	-0.034	0.007	-0.078	0.021	-0.090	-0.030	-0.074	-0.101	-0.092	-0.179	-0.149	-0.117	-0.101	-0.
Q33	-0.030	0.076	0.015	-0.037	-0.029	0.009	-0.007	-0.021	-0.048	-0.048	-0.040	0.004	0.001	-0.035	-0.007	-0.040	-0.039	-0.048	0.007	0.
Q34	0.046	-0.019	-0.034	-0.044	-0.042	0.035	-0.034	-0.044	-0.047	-0.050	-0.057	-0.046	-0.075	-0.104	-0.081	-0.146	-0.150	-0.156	-0.039	-0.
Q35	-0.030	-0.049	-0.042	-0.018	-0.023	0.032	-0.002	0.028	-0.093	-0.025	-0.033	0.155	-0.011	-0.080	-0.078	-0.127	-0.140	-0.143	-0.060	-0.
Q36	-0.045	-0.035	-0.067	-0.061	-0.072	-0.073	-0.052	-0.061	-0.089	-0.022	-0.110	-0.071	0.006	-0.066	-0.096	-0.153	-0.152	-0.145	-0.105	-0.
Q37	-0.092	-0.019	-0.121	-0.047	-0.058	-0.115	-0.053	-0.081	0.015	-0.057	-0.028	-0.028	0.030	-0.122	-0.155	-0.097	-0.085	-0.085	-0.002	0.
Q38	-0.034	-0.017	-0.071	-0.039	-0.035	-0.095	-0.018	-0.035	-0.020	-0.058	-0.065	-0.029	0.015	-0.082	-0.058	-0.084	-0.095	-0.043	0.055	-0.

[3]

Item Statistics

	Proportion	Measure	S.E.Measure	Infit	Outfit
Q1	0.870	-2.374	0.0688	0.898	0.678
Q2	0.969	-4.214	0.1301	0.824	0.411
Q3	0.598	-0.485	0.0483	1.121	1.213
Q4	0.932	-3.232	0.0904	0.869	0.532
Q5	0.927	-3.137	0.0875	0.885	0.805
Q6	0.665	-0.853	0.0500	1.041	1.061
Q7	0.943	-3.462	0.0981	0.890	0.634
Q8	0.875	-2.426	0.0698	0.860	0.643
Q9	0.840	-2.074	0.0633	1.142	1.339
Q10	0.879	-2.471	0.0707	1.047	1.336
Q11	0.604	-0.518	0.0484	0.987	0.966
Q12	0.706	-1.099	0.0517	0.983	0.961
Q13	0.930	-3.191	0.0891	0.929	0.722
Q15	0.766	-1.483	0.0553	0.927	0.847
Q16	0.791	-1.671	0.0575	0.888	0.795
Q17	0.563	-0.301	0.0477	0.890	0.838
Q18	0.546	-0.215	0.0475	0.882	0.827
Q19	0.479	0.131	0.0473	0.961	0.940
Q21	0.892	-2.634	0.0743	0.940	0.907
Q22	0.115	2.526	0.0703	1.060	1.296
Q23	0.383	0.631	0.0484	1.012	0.993
Q25	0.595	-0.469	0.0482	1.127	1.185
Q26	0.749	-1.370	0.0541	0.965	0.909
Q27	0.604	-0.518	0.0484	1.035	1.032
Q28	0.238	1.488	0.0543	1.076	1.322
Q29	0.479	0.133	0.0473	1.074	1.120
Q30	0.898	-2.707	0.0760	1.031	1.338
Q31	0.829	-1.980	0.0618	1.068	1.220
Q32	0.706	-1.099	0.0517	1.081	1.195
Q33	0.971	-4.284	0.1337	0.826	0.588
Q34	0.643	-0.727	0.0493	0.964	0.910
Q35	0.892	-2.628	0.0741	0.933	0.848
Q36	0.593	-0.459	0.0482	1.073	1.078
Q37	0.606	-0.527	0.0484	1.071	1.082
Q38	0.385	0.622	0.0484	1.074	1.116

Note. Infit= Information-weighted mean square statistic; Outfit= Outlier-sensitive means square statistic.

[3]

Wright Map

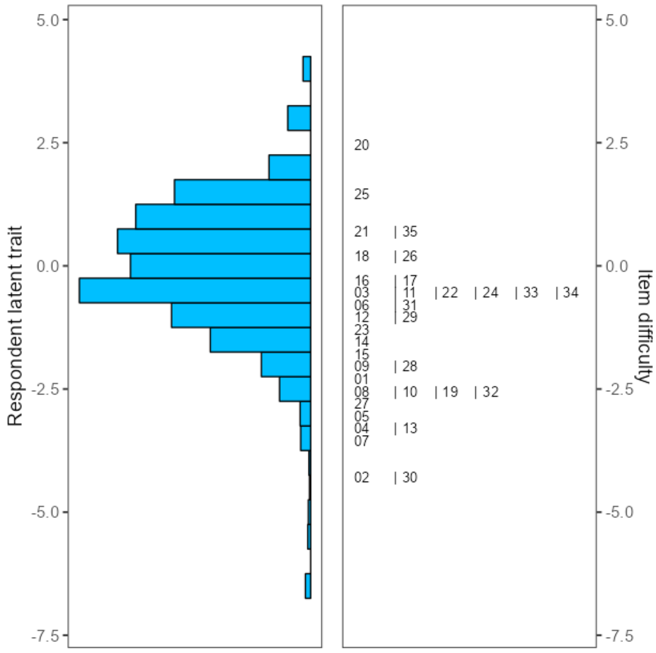

[4]

Expected Score Curve

Q1

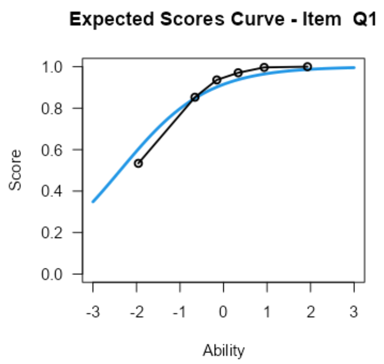
Expected Scores Curve - Item Q1
Score
Ability
1.0
0.8
0.6
0.4
0.2
0.0
-3
-2
-1
0
1
2
3

Q2

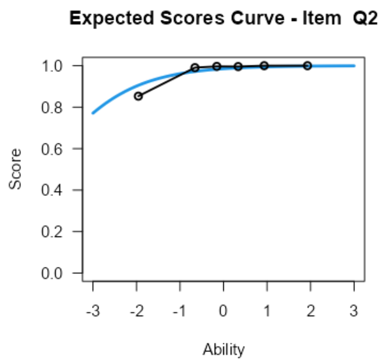
Expected Scores Curve - Item Q2
Score
Ability
1.0
0.8
0.6
0.4
0.2
0.0
-3
-2
-1
0
1
2
3

Q3

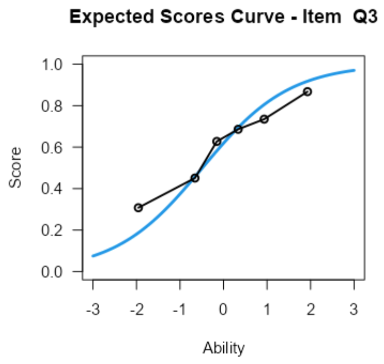
Expected Scores Curve - Item Q3
Score
Ability
1.0
0.8
0.6
0.4
0.2
0.0
-3
-2
-1
0
1
2
3

Q4

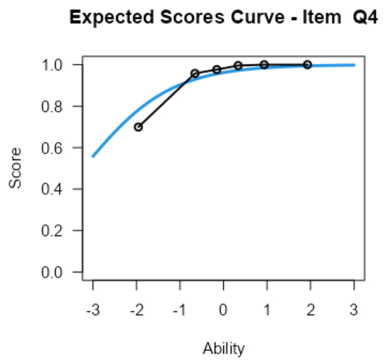
Expected Scores Curve - Item Q4
Score
Ability
1.0
0.8
0.6
0.4
0.2
0.0
-3
-2
-1
0
1
2
3

Q5

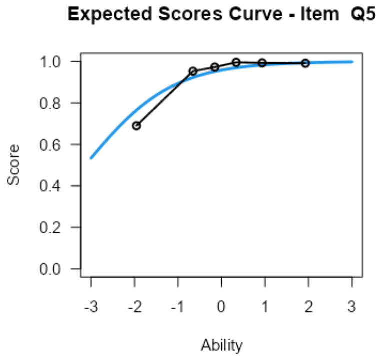
Expected Scores Curve - Item Q5
Score
Ability
1.0
0.8
0.6
0.4
0.2
0.0
-3
-2
-1
0
1
2
3

Q6

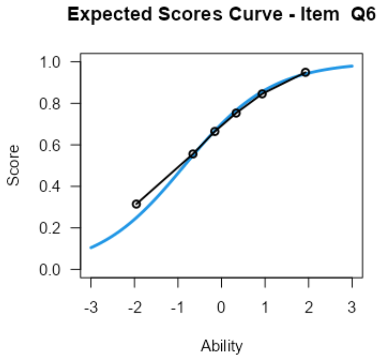
Expected Scores Curve - Item Q6
Score
Ability
1.0
0.8
0.6
0.4
0.2
0.0
-3
-2
-1
0
1
2
3

Q7

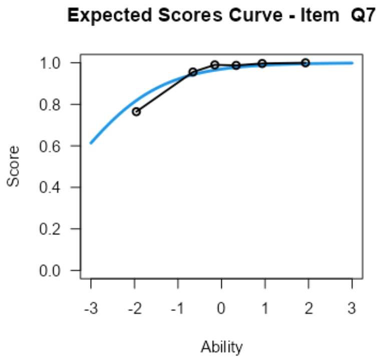
Expected Scores Curve - Item Q7
Score
Ability
1.0
0.8
0.6
0.4
0.2
0.0
-3
-2
-1
0
1
2
3

Q8

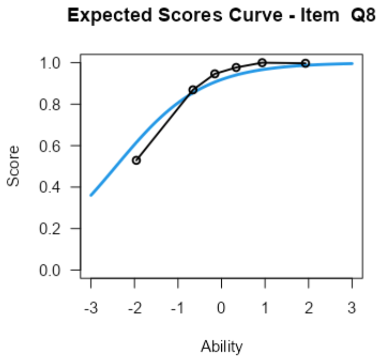
Expected Scores Curve - Item Q8
Score
Ability
1.0
0.8
0.6
0.4
0.2
0.0
-3
-2
-1
0
1
2
3

Q9

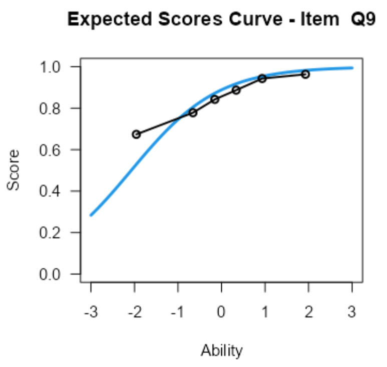

Q10

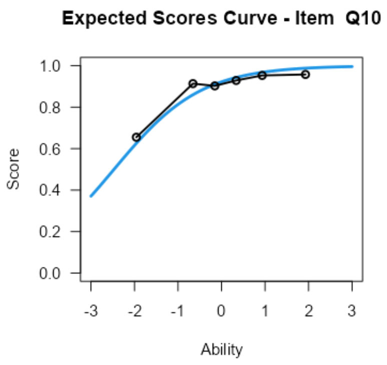

Q11

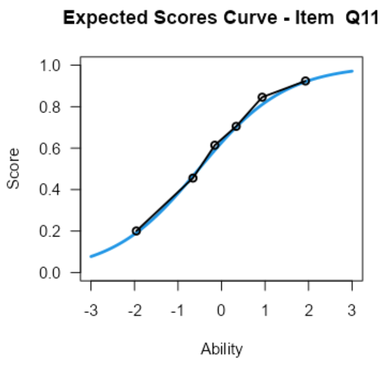

Q12

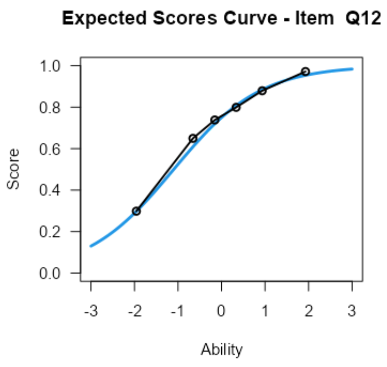

Q13

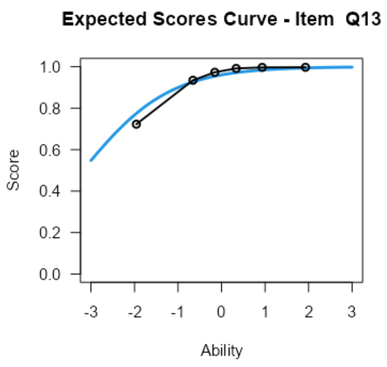

Q15

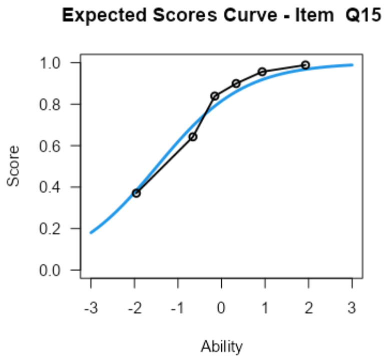

Q16

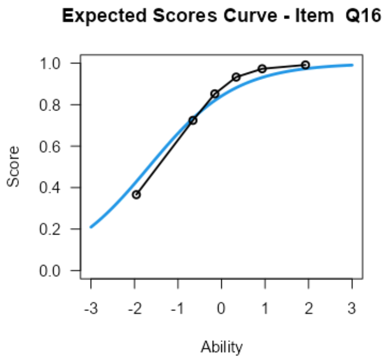

Q17

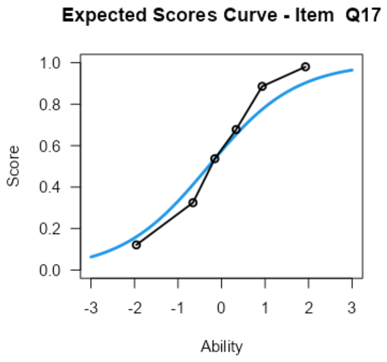

Q18

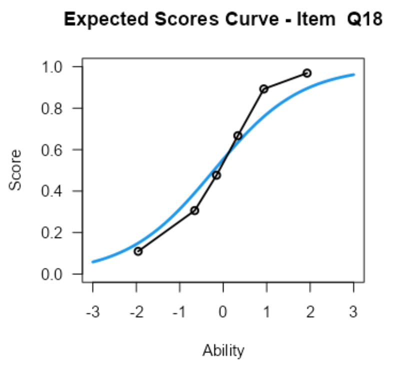

Q19

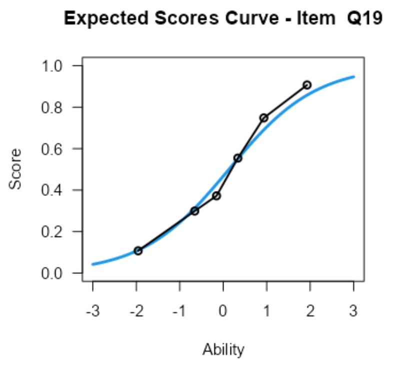

Q21

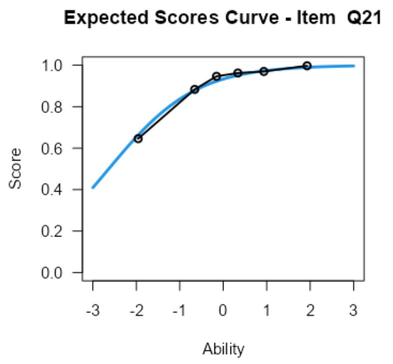

Q22

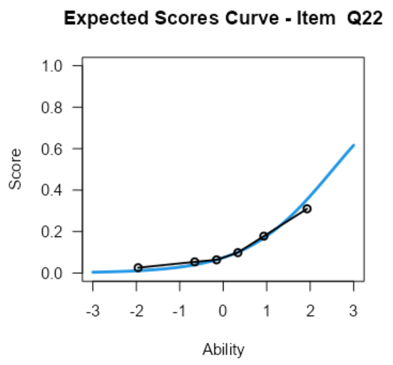

Q23

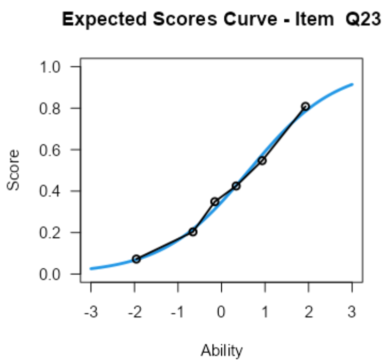

Q25

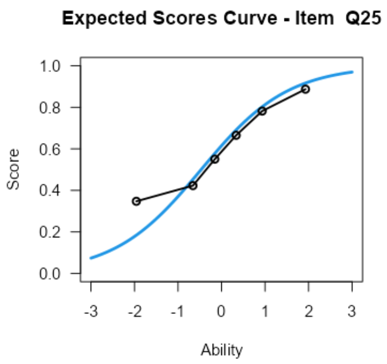

Q26

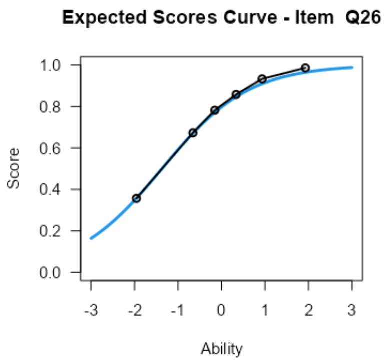

Q27

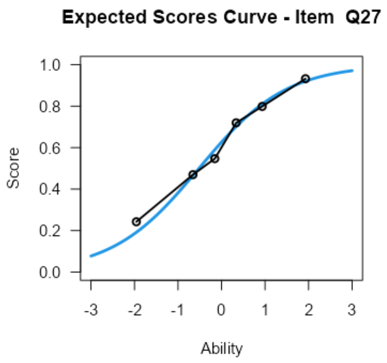

Q28

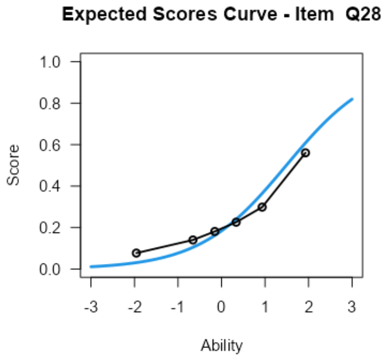

Q29

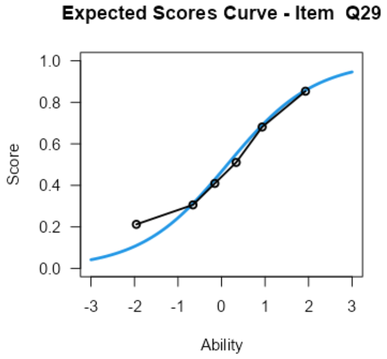

Q30

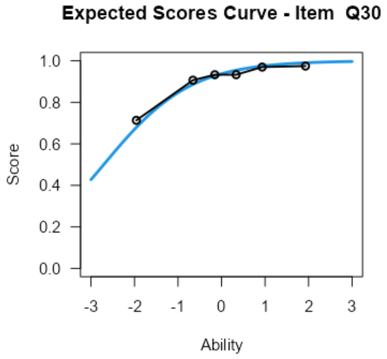

Q31

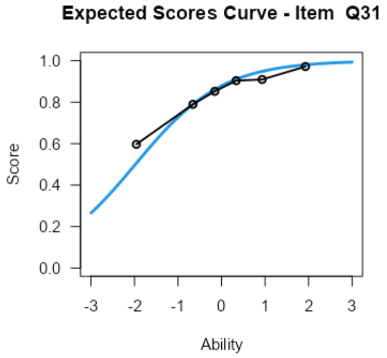

Q32

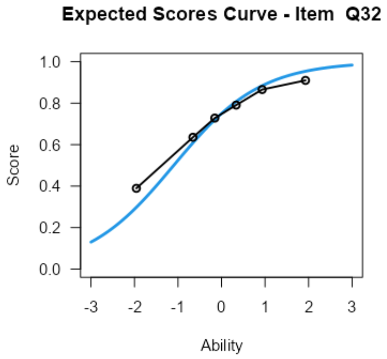

Q33

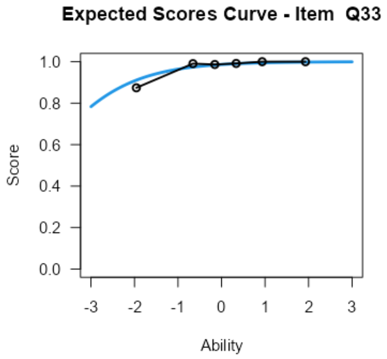

Q34

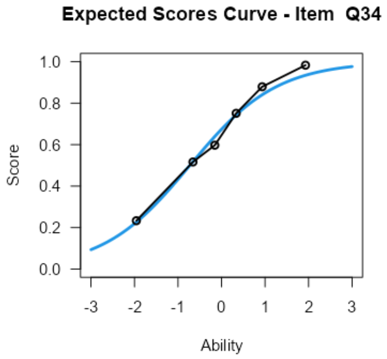

Q35

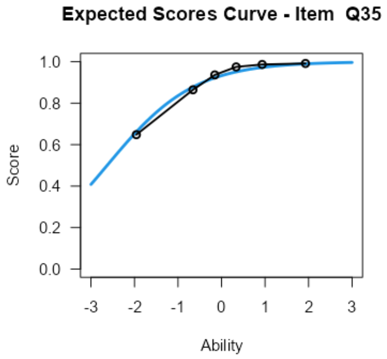

Q36

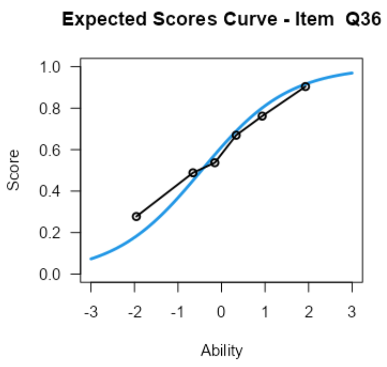

Q37

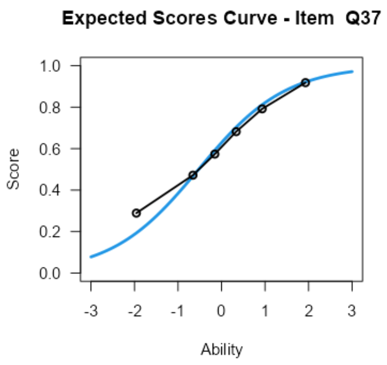

Q38

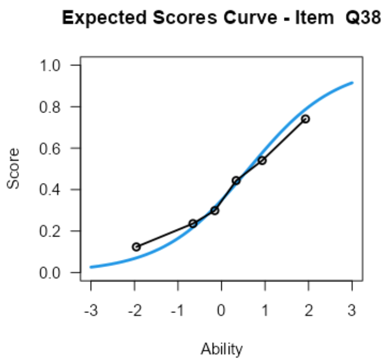

[5]

References

[1] The jamovi project (2022). *jamovi*. (Version 2.3) [Computer Software]. Retrieved from https://www.jamovi.org.

[2] R Core Team (2021). *R: A Language and environment for statistical computing*. (Version 4.1) [Computer software]. Retrieved from https://cran.r-project.org. (R packages retrieved from MRAN snapshot 2022-01-01).

[3] Robitzsch,A., Kiefer, T., & Wu, M. (2020). *TAM: Test Analysis Modules*. [R package]. Retrieved from https://CRAN.R-project.org/package=TAM.

[4] Martinkova, P., & Drabinova, A. (2018). *ShinyItemAnalysis: for teaching psychometrics and to enforce routine analysis of educational tests*. [R package]. Retrieved from https://CRAN.R-project.org/package=ShinyItemAnalysis.

[5] Seol, H. (2020). *snowIRT: Item Response Theory for jamovi*. [jamovi module]. Retrieved from https://github.com/hyunsooseol/snowIRT.
